# Rgg/SHP transcriptional regulators, RaS-RiPPs, and their impacts in streptococci

**DOI:** 10.12688/f1000research.178446.1

**Published:** 2026-03-19

**Authors:** Sristi Dey, Sophie A. Krivograd, Britta E. Rued

**Affiliations:** 1 Veterinary Microbiology and Preventive Medicine, Iowa State University College of Veterinary Medicine, Ames, Iowa, 50010, USA

**Keywords:** Streptococci, Rgg/SHP, competence, quorum sensing, RaS-RiPPs

## Abstract

Streptococci are prevalent in animal and human microbiomes. These organisms produce a vast array of small peptides that modulate complex functions within the cell such as quorum sensing, virulence, and metabolism. Transcriptional regulators are central to this process, of which Rgg transcriptional regulators hold prominence in streptococci. These systems are controlled by peptides known as SHPs (short hydrophobic peptides) and LCPs (leaderless communication peptides). Also known as Rgg/SHP quorum sensing (QS) systems, they are ubiquitous across streptococcal species and regulate cellular competence, metabolic programs, virulence, and facilitate colonization of host species. It has been recently demonstrated that Rgg/SHP QS systems can also regulate the production of natural products known as RaS-RiPPs ( **Ra**dical ***S***-adenosylmethionine enzyme **Ri**bosomally translated and **P**ost-translationally modified **P**eptides). RaS-RiPPs are widespread in streptococci with sixteen current subfamilies. Some of these natural products possess inhibitory properties while others’ functions are currently unknown. We provide here a review of Rgg/SHP systems within streptococci, the complexities and characterized functions of RaS-RiPPs, as well as the connection between Rgg/SHP and RaS-RiPPs.

## Introduction

Bacterial species respond to different stimuli essential for survival through cell density-linked signaling circuits called quorum sensing (QS) systems. These systems synchronously control gene expression via the detection and processing of chemical signals and cognate receptors. In response to cell density, QS systems control cell-cell communication via the production of “autoinducers” or “pheromones” synthesized intracellularly. ^1, 2^ These systems have been demonstrated to control cellular processes involved in colonization, virulence, biofilm formation, and important metabolic programs. ^1, 3– 11^ Gram-positive species possess these systems in abundance and utilize RRNPP transcriptional regulators to control these systems. The RRNPP family, standing for Rap, **r**esponse regulator **a**spartate **p**hosphatase; Rgg, **r**egulator **g**ene of **g**lucosyltransferase; NprR, **n**eutral **pr**otease **r**egulator; PlcR, **p**hospho **l**ipase **C r**egulator; and PrgX, **p**heromone- **r**esponsive **g**ene **X**; are united by the fact that they are transcriptional regulators that respond to a small autoinducer. ^12– 14^ Rggs are some of the primary regulators of quorum sensing systems in streptococci. ^2, 12, 13, 15, 16^ These proteins interact with cognate peptide pheromones via a ~ 220-residue tetratricopeptide repeat (TPR) and bind to genes that they regulate using a 60-residue helix-turn-helix (HTH) domain. ^9, 17^ The peptide pheromones that the TPR domain interacts with are called SHPs ( **s**hort **h**ydrophobic **p**eptides), and as more recently demonstrated LCPs ( **l**eaderless **c**ommunication **p**eptides). ^7, 8, 11, 13, 18– 20^ SHPs are often encoded directly next to genes that produce the Rgg transcriptional regulator, ^2, 3, 5, 7, 21– 24^ and contain an abundance of hydrophobic amino acids such as leucine, isoleucine, valine, and glycine. ^3, 25, 26^ The binding of a SHP peptide to its cognate Rgg results in a conformational shift, ^20^ and thus regulation of promoters at which the Rgg binds. This can result in transcriptional activation or repression, depending on the Rgg. ^3^ SHP peptides are essential for triggering this process and their sequence is Rgg system specific. ^3, 7, 11, 27– 30^ Classifications for these SHPs have been proposed based on the transcriptional orientation of SHPs and Rggs and the amino acid content of SHP peptides. ^31^ In this classification, Group I and II SHPs are divergently transcribed from the cognate Rgg regulator, and the SHP peptide contains an N-terminal aspartate or glutamate residue, while Group III SHPs overlap with their cognate Rgg gene at which they are convergently transcribed from. However, as previously mentioned, it was recently discovered that there is another family of short peptides that bind to Rgg regulators in streptococci. These do not fit into the aforementioned classification of SHP peptides, as they have characteristics that make them distinct. This subset is composed of leaderless peptides, encoding for a mature amino acid without the leader sequences present in SHPs that are necessary for processing and secretion. These have been named LCPs and are widespread across streptococci and Firmicutes. ^32^ The first LCP to be discovered was found in *Streptococcus pyogenes*, in which SIP (SpeB-inducing peptide) activates the Rgg regulator also known as RopB. ^11^ Similar to SHPs, SIP is divergently transcribed from RopB. ^11^ As such, peptides involved in streptococcal QS can be split into SHPs and LCPs.

Orthologs of Rgg proteins are widespread in low G + C Gram-positive bacteria, ^3, 5, 14, 16^ but Rgg/SHP regulators are concentrated in streptococcal species. They have been demonstrated to be involved in virulence, biofilm formation, colonization, and metabolism, among other functions. Multiple studies have demonstrated their importance in streptococci such as *S. pneumoniae, S. mutans*, *S. pyogenes*, *S. thermophilus, S. agalactiae, S. salivarius* and others. ^5, 6, 9, 17, 33, 34^ The transcriptional regulation of these systems varies from species to species and targets unique loci depending on the streptococcal organisms they are present in. Of these targets, RaS-RiPPs ( **Ra**dical ***S***-adenosylmethionine enzyme and **Ri**bosomally translated and **P**ost translationally modified **P**eptides) have emerged as loci regulated by Rgg/SHPs. ^23, 30, 35– 37^ These systems encode small ribosomally translated peptides that are post-translationally modified by a radical *S*-adenosylmethionine (SAM) enzyme, ^23, 35^ and are typically secreted into the extracellular space. They have been shown to inhibit other streptococcal species and have varying effects on antibiotic resistance and growth. ^30, 38^ As such, in the past eight years these have begun to emerge as a suite of biosynthetic gene operons that are controlled by Rgg/SHP systems. This review aims to provide an overview of Rgg/SHP systems among streptococcal species, their connection to RaS-RiPP systems, as well as their effects on streptococcal physiology themselves.

### Streptococcus pneumoniae

Cell-cell communication systems have been extensively studied in *S. pneumoniae,* outside of Rgg/SHP systems. The best characterized by far is the ComCDE system that induces competence, which we will briefly discuss later. These have been the subject of intense study for approximately a century, dating back to the initial studies by Frederick Griffith in 1928 demonstrating that a transforming principle existed in *S. pneumoniae* that could lead to the metamorphosis of avirulent rough strains to virulent smooth strains in mice. ^39^ In *S. pneumoniae,* this system is controlled via ComCDE. The secreted pheromone, **c**ompetence **s**timulating **p**eptide (CSP) is produced from ComC precursor peptide. ComC carries a double-glycine leader sequence required for export and cleavage by ComAB transporter to produce mature peptide. ^40, 41^ Extracellular CSP binds with ComD, a histidine kinase regulator that subsequently activates ComE to produce early genes through a positive feedback loop. ^40, 42– 44^ This then results in activation of the alternative sigma factor *sigX* (also known as *comX*), which is responsible for the production of genes that allow for the development of competence. ^45– 48^ Many studies have been written on this subject and we briefly summarize this system later on in this review, along with referring the reader to several reviews on the topic.

**Figure 1.**
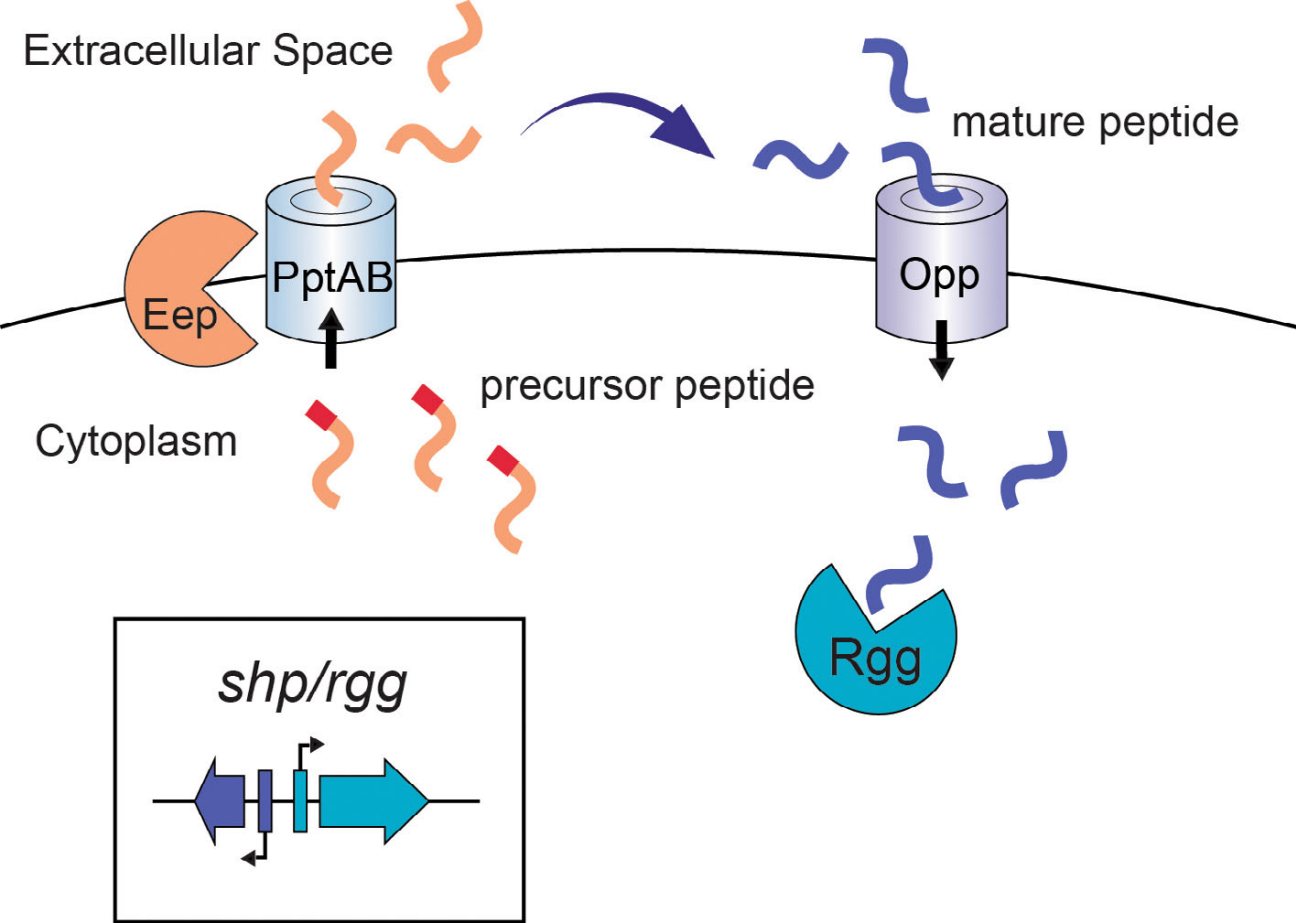
Overview of Rgg/SHP quorum sensing. Precursor peptides are trimmed by Eep or alternative peptidases. They require transport through PptAB to the extracellular space. Maturation of the SHP is known to occur in some cases in the extracellular space or during transport. The mature peptide is transported back into the cytoplasm through the Opp transporter where it can bind to Rgg, which induces downstream genes and increased production of the cognate SHP.

In terms of Rgg/SHP quorum sensing systems, the study of these in pneumococci dates to only the past decade. Several Rgg/SHP systems have been found in *S. pneumoniae.* These have been primarily named based on the locus number assigned to the Rgg of interest and include: Rgg144/SHP144, Rgg939/SHP939, RtgR/RtgS, and Rgg1518/SHP1518 ( Table 1). One of the first Rgg/SHP quorum sensing system characterized in *S. pneumoniae* was the Rgg939/SHP939 system. ^6^ Like other Rgg/SHP systems, it consists of an Rgg transcriptional regulator (Rgg939) and a short hydrophobic peptide (SHP939). The precursor SHP is synthesized within the cell and secreted through a peptide transporter called PptAB, and processed via a membrane protease called Eep, as is seen in most Rgg/SHP systems ( Figure 1). ^29, 49^ When peptide densities increase, the mature SHP is imported into the cell by the oligopeptide permease (Opp) transporter, which then binds to Rgg939 to activate gene expression. This in turn drives the expression downstream genes via a positive feedback loop. ^3, 29, 33^ The Rgg939/SHP939 signaling system induces the expression of 11 genes present in a single transcript downstream of *shp* that comprises variety of essential functions such as *mnaB*, UDP-4-galactose-epimerase, a putative xylose isomerase, an AMP-binding enzyme, lantibiotic and bacitracin transport, lanthionine biosynthesis protein, as well as a lactose transporter. Expression of these genes influences polysaccharide production. Additionally, a *S. pneumoniae* D39 strain that lacks this Rgg has impaired biofilm formation and lower fitness in a murine model of lung infection. ^6^

**Table 1.** Functional streptococcal SHP and XIP peptides.

Transcrip. Reg. ^a^	SHP/XIP sequence	Peptide Name ^b^	Genome	Ref. ^c^
***S. pneumoniae***				
Rgg144	VIPFLTNL	SHP144	*S. pneumoniae* D39	^7, 31^
Rgg939	DIIIIVGG	SHP939	*S. pneumoniae* R6, D39	^6^
Rgg1518	IQLIWFETWFWG	SHP1518	*S. pneumoniae* D39	^27^
RtgR	AIIFPWGWPI	RtgS1 Type A	*S. pneumoniae* D39, SP-BS68	^19^
RtgR	AIIFPWGWSI	RtgS1 Type B	*S. pneumoniae* D39	
***S. pyogenes***				
Rgg2	DILIIVGG	SHP2	*S. pyogenes* NZ131	^18^
Rgg3	DIIIIVGG	SHP3	*S. pyogenes* NZ131	
RopB	MWLLLLFL	SIP	*S. pyogenes* MGAS10870	^11, 78^
ComR	SAVDWWRL	M1 XIP	*S. pyogenes* M1 SF370, MGAS8232, MGAS10394, MGAS6180, MGAS5055, MGAS9429, ATCC 10782, MGAS2096, MGAS10750, NZ131	^22, 131^
ComR	EFDWWNLG	M3 XIP	*S. pyogenes* Manfredo, MGAS10270, MGAS315, SSI-1	
***S. mutans***				
Rgg1509	ETIIIIGGG	SHP1509	*S. mutans* UA159	^25^
PdrA (SMU_1509)	ETIIIIGG	SHP	*S. mutans* UA159	^30^
ComR	GLDWWSL	XIP	*S. mutans* UA159	^30, 102^
***S. ferus***				
ComR	GLSWWGL	XIP	*S. ferus* DSM20646	^86^
***S. thermophilus***				
Rgg1358	EGIIVIVVG	SHP1358/SHP768	*S. thermophilus* LMD-9	^25, 89^
Rgg1299	DIIIFPPFG	SHP1299/SHP714	*S. thermophilus* LMD-9	^25^
Rgg9420	EGIIVIGVG	SHP279	*S. thermophilus* LMD-9	^89^
Rgg _Sthermo_6_	DIIIFPPFG	SHP _Sthermo_6_	*S. thermophilus* ST13	^37^
Rgg _Sthermo_12_	DIIIIVGG	SHP _Sthermo_12_	*S. thermophilus* STH_CIRM_1047	
Rgg _Sthermo_13_	EGIIVIGVG	SHP _Sthermo_13_	*S. thermophilus* TSGB 4234	
Rgg _gp_sali_3_	CIYTIVGGV	SHP _gp_sali_3_	*S. thermophilus* STH_CIRM_1125, CNRZ1066, ena-SAMPLE-787-33427	
Rgg _gp_sali_4_	EIIIIIAL	SHP _gp_sali_4_	*S. thermophilus* STH_CIRM_1121, MV-FAST4, JIM8232	
Rgg _gp_sali_5_	ESIIVIAVG	SHP _gp_sali_5_	*S. thermophilus* St ^−10^, Vach60, JIM8232, CNRZ1066	
Rgg _gp_sali_6_	EGIIVIVVG	SHP _gp_sali_6_	*S. thermophilus* TH1447, ST057-1, TSGB 4243, Vach60, JIM8232.	
ComR	LPYFAGCL	XIP	*S. thermophilus* LMD-9	^132, 133^
***Streptococcus mitis***				
Rgg0094	DIIIVGG	SHP0094	*S. mitis* CCUG31611	^26^
***Streptococcus suis***				
ComR	GNWGTWVEE	Type A XIP	*S. suis* SS2 Chz, ZY05719	^134^
ComR	GNWGKWTDG	Type B XIP	*S. suis* CZ130302	
ComR	LGDENWWVK	Type C XIP	*S. suis* ZJJX0908005	
**Other streptococcal species**				
RovS	DILIIVGG	SHP1520	*S. agalactiae* NEM316/A909	^15, 25^
Rgg2	DILIIVGG	SHP2	*S. dysgalactiae* subsp. e *quisimilis* GGS-LT1	^15^
Rgg	LLLLKLA	SHP	*S. zooepidimicus* ATCC35246	^8^
ComR	VPFFMIYY	XIP _Sve_	*S. vestibularis*	^132, 135^
ComR	LMCTIAR, LMCTIVR	XIP	*S. sobrinus* NIDR 6715-7, NCTC 10919	^136^
ComR	LTAWWGL	_Sin_ComS _9-15_	*S. infantarius* AV2A, 3AG-1, 11FA-1, ATCC BAA-102, CJ18	^105^
ComR	ITGWWGL	_Sma_ComS _9–15_	*S. macedonicus* DSM15879, ACA-DC198, 679	
ComR	LPYFAGCL	sXIP	*S. salivarius* HSISS4	^135^

^a^ Transcrip. Reg. stands for transcriptional regulator.

^b^ Peptide sequences follow naming conventions as presented in original publications.

^c^ Ref. stands for reference.

At the same time that the Rgg939/SHP939 system was discovered, another Rgg/SHP was found in *S. pneumoniae.* This was named Rgg144/SHP144. Rgg144 as well as Rgg939 can induce QS in response to sugars found in the respiratory tract, such as galactose and mannose, alongside their native SHP induction. Rgg144 and Rgg939 perform some level of crosstalk, with the presence of Rgg939 and Rgg144 system necessary for full QS induction of each other. Rgg144 and Rgg939 are both important for processes such as mannose metabolism, as well as necessary for pneumococcal colonization in the nasopharynx. ^7^ Rgg144 is also vital for production of a short peptide called the VP1, which has been demonstrated to play a role in pneumococcal colonization and virulence. ^7, 28, 50^

The Rgg1518/SHP1518 system is another Rgg/SHP system that has been characterized in *S. pneumoniae.* This system has also been implicated in pneumococcal virulence and is responsive to sugars such as galactose and mannose, a common theme in *S. pneumoniae* Rgg/SHP systems. Strains that lack this Rgg have lower growth yields and extended lag phases when grown on galactose and mannose as primary carbon sources. This Rgg is also a regulatory nexus, at which Rgg144, Rgg939 and another QS regulator TprA function to control of sugar transport, galactose metabolism and capsule synthesis. ^6, 7, 27^ TprA and its cognate peptide PhrA are distinct QS regulatory systems from Rgg/SHP systems but has also been shown to have impacts on sugar metabolism, neuraminidase activity, lantibiotic expression, and virulence. ^51– 53^ In this regulatory interaction, Rgg144 and Rgg939 also impact the transcription of Rgg1518. Rgg1518 acts as a negative repressor of capsule synthesis, as deleting this gene results in increased capsule polysaccharide in the presence of galactose. Finally, it has been demonstrated to directly impact pneumococcal colonization, as a deletion of *rgg1518* results in significantly lower CFU/mL in the nasopharynx of a murine pneumococcal colonization model. ^27^

Finally, RtgR/RtgS (hereafter RtgR/S) is an Rgg/SHP-like system found in pneumococcus that impacts nasopharyngeal colonization. This system belongs to the same family of regulatory systems as Rgg/SHP and ComR/S system found in streptococci and controls the expression of the *rtg* locus ( **R**gg-regulated **t**ransporter of double glycine peptides) which encodes for peptidase-containing ABC transporters (PCAT) and *rtgS.* RtgS is similar to SHPs and XIPs (peptides involved in induction of competence in streptococci) in most aspects except it lacks a conserved aspartate or glutamate residue. The presence of this system in pneumococci has been demonstrated to confer a survival advantage: wild-type strains with RtgR/S outcompeted strains that lacked the system during nasopharyngeal colonization in mice. ^5, 19^

Thus, *S. pneumoniae* harbors several Rgg/SHP-like transcriptional regulatory systems that play important roles in pneumococcal physiology such as virulence, colonization, biofilm formation, and sugar metabolism. Some of the Rgg/SHP regulators have interlinked functions possessing cognate pheromone inducers present in the core and accessory genome driving specific functions that integrate metabolic state and environmental or fitness cues. Together, these Rgg systems illustrate that pneumococcus has a diverse set of Rgg-like transcriptional quorum sensing systems to adapt to host niches and coordinate community behaviors.

### Streptococcus pyogenes

*S. pyogenes* was one of the first organisms in which Rggs were demonstrated to interact with SHP peptides to act as transcriptional regulators, ^3^ aside from *S. thermophilus.* In *S. pyogenes,* the first study to examine this compared Rgg transcriptional regulators in *S. pyogenes* and identified two of these Rggs as having a high-level of similarity to each other (55% identical, 76% similar). Upon examining the coding region around the Rgg regulators, small, unannotated ORFs that were predicted to encode for 22 and 23 amino acids were identified. Further experimental validation demonstrated that these encoded for **s**hort **h**ydrophobic **p**eptides (SHPs), later renamed as SHP2 and SHP3 ( Table 1) based on their proximity to their specific Rgg regulators. These systems are essential for the induction of target genes and together Rgg/SHPs composed a functional quorum sensing system in *S. pyogenes.* ^31^ Expression of SHPs in *S. pyogenes* NZ131 requires a functional Rgg2, whereas Rgg3 ( Table 1) acts as a transcriptional repressor. ^3^ In this system, SHP pheromones (DI [I/L]IIVGG) require a functional oligopeptide permease ( *opp)* transporter and a metalloprotease ( *eep)* to export precursor peptides and enzymatically mature them. The pro-peptides for SHPs are converted to mature peptides, SHP2-C8 and SHP3-C8, which can then be imported back into the cytoplasm in an Opp-dependent manner to bind to Rgg regulators and drive transcription. ^15, 54– 57^ Rgg2 and Rgg3 have been shown to have differential activation of Rgg target genes, including a large biosynthetic operon of unknown function and a locus encoding for a small protein called *stcA.* ^58, 59^ Rgg2 activates *shp* expression and regulated loci, whereas Rgg3 represses expression of the system by forming an opposing regulatory circuit. ^3^ Rgg2 and Rgg3 have a competitive relationship due to highly conserved overlapping promoter binding sites present in both the *shp2* and *shp3* promoters. When SHPs bind Rgg2 this drives the activation of quorum sensing, but during SHP-limited conditions, Rgg3 predominantly maintains the system in an inactive state. ^20, 60^ Later structural analyses of Rgg2 and Rgg3 revealed that both proteins could act as transcriptional activators under specific conditions, such as highly increased SHP concentrations. Therefore, Rgg3 is not strictly repressive by mechanism; but acts as a repressor during low-SHP conditions. ^20^

One of the main targets of the Rgg2/Rgg3 system is the gene *stcA.* StcA acts as a cell wall binding protein, confers lysozyme resistance and is necessary for *S. pyogenes* biofilm formation. StcA is secreted, binds to peptidoglycan in the cell wall via electrostatic interactions, and as such localizes to the cell surface. StcA is also thought to potentially function in conjunction with putative S-layer transglutaminases in the cell to form an S-layer, although this has yet to be definitively determined. ^58^ The other target of Rgg2/Rgg3 signaling is a large biosynthetic gene operon, which was recently renamed *qim* ( **q**uorum-regulated **i**mmunomodulatory **m**odification). ^61^ This operon and *stcA* are upregulated during murine skin infection and murine nasal associated lymphoid tissue (NALT) colonization. ^58, 61, 62^ A recent report demonstrated that *qim* modifies the cell wall of *S. pyogenes* by adding a unique *N*-acetylglucosamine-linked ribitol that suppresses the innate immune response via an unknown mechanism. The presence of this modification is necessary for full virulence in a murine skin model, as well as preventing NF-κB activation. ^63^

The activation of the Rgg2/Rgg3 quorum sensing pathway and the genes that it regulates helps to provide a survival advantage to *S. pyogenes* during colonization. In a murine skin infection model with QS-active (WT and ∆ *rgg3*) strains, the presence of this system results in significant weight loss, greater bacterial burden, and progressive loss of epithelial barrier integrity with lesions. Compared to QS-active mutants, when mice were infected with QS-null mutant (∆ *rgg3*shp2 _GGG_shp3 _GGG_) strains started developed crusted lesions, clearing central erythema and had continuous healing from day 5 to 10 post-inoculation. These results demonstrated that an intact Rgg2/Rgg3 system provides advantages for the survival of *S. pyogenes* on skin infection. ^61^ This system also impacts colonization in a murine oropharyngeal model. Constitutive expression of the system results in higher levels of colonization in mice and lower expression of regulatory cytokines. In contrast, deletion of the positive regulator Rgg2 (thus inactivation of the system) cannot establish colonization in mice. ^62^

Other evidence has shown that this system is important for virulence gene expression as well. Deletion of Rgg2 in the M1 serotype results in differential expression of several virulence factors: lower expression of SIC (streptococcal inhibitor of complement), a streptococcal exotoxin H precursor, and higher expression of genes such as *slo, nga*, and *scpA.* Rgg2 deletion also results in attenuation of *S. pyogenes* in an intraperitoneal murine model. ^64^ In *S. pyogenes* NZ131, induction of this system leads to the lower expression of *slo* (streptolysin O) due to increased expression of the *spy49_0460* efflux protein, in agreement with observations from M1 serotype in its absence. Proteins such as SpyCEP and M protein had decreased expression when the Rgg2/Rgg3 system was active. Thus, it appears that the Rgg2/Rgg3 system is necessary for *S. pyogenes* colonization and correct expression of virulence factors. ^59^

In line with the necessary requirement for Rgg2/Rgg3 for full virulence and colonization, the Rgg2/Rgg3 system suppresses macrophage responses and pro-inflammatory immune responses. Infection of macrophages with a functional Rgg2/Rgg3 system or the system in a QS locked on state suppresses macrophage NF-kB activity, TNF-α, and IL-6 production. This process necessitates live cells, is thought to be an active process, and requires the presence of the *qim* operon. ^65^ Macrophages infected with QS-locked on strains downregulate inflammatory pathways and upregulate fatty acid beta-oxidation and oxidative phosphorylation pathways in M2 macrophages. Further investigation of this phenotype found that suppression of inflammatory responses via Rgg2/Rgg3 are primarily due to epigenetic regulation and disruption of transcription factor translocation to the nucleus. ^66^

While SHPs are the main way the Rgg2/Rgg3 system is induced, it can also be triggered via metal availability, different carbon sources, and nitric oxide (NO). ^67^ MtsR, a DtxR-family metallorepressor, binds upstream of *shp3* in response to low iron and manganese levels and represses transcription. ^68, 69^ Mannose availability also impacts the Rgg2/Rgg3 system, but this is modulated in NZ131 by the Mga transcription regulator and a mannose PTS system (PtsABCD). NO triggers Rgg2/Rgg3 system induction via formation of dinitrosyliron complexes (DNIC) resulting in NO-dependent iron restriction. ^67^ Hence, its involvement is also linked to the response to low iron conditions. As such, metal and carbon sensing are distinct regulatory systems that converge during SHP pheromone production and Rgg2/Rgg3 activation. ^70– 72^

RopB (Regulator of Protease B, *spy49_1691*, also called Rgg) is another Rgg present in *S. pyogenes.* RopB represents a unique class of Rgg regulators that respond to LCPs, termed SIPs in *S. pyogenes.* ^54– 56^ RopB is located adjacent to *speB,* and is required for the activation of *speB,* which encodes the extracellular cysteine protease of streptococcal erythrogenic toxin B. ^73^ RopB directly controls the expression of *speB* by binding to operator elements at the intergenic region between the *ropB* and *speB* transcription start site and drives the transcription of *speB* in a growth-phase dependent manner. ^11, 73, 74^ RopB also impacts expression of the autolysin *clpB*, a DNA entry nuclease. ^74^ Due to the targets it regulates, RopB is also important for virulence and colonization. For instance, presence of this system is vital for colonization of the mouse oropharynx, survival in blood, and full virulence. ^11, 75– 79^ Another small peptide besides SIP has been demonstrated to impact RopB activity, called Vfr. Vfr acts as an inhibitory peptide and is thought to interact with RopB, preventing it from binding DNA and thus repressing *speB* transcription. ^11, 73– 75, 77, 78, 80^

The discovery of the Rgg2/Rgg3 system established the use of SHPs as QS signals outside of *S. thermophilus*, while the finding that RopB utilizes a distinct LCP called SIP expanded the field’s understanding of peptide signaling in streptococci. Much of the field’s current understanding of Rgg signaling has been established via the study of these systems in *S. pyogenes* and *S. thermophilus,* as we discuss later, which has involved the contribution of multiple groups.

### Streptococcus gordonii

While Rgg/SHP systems as quorum sensing systems were established in *S. pyogenes* and *S. thermophilus,* ^3, 5^ Rgg proteins themselves had already been defined as transcriptional regulators. In fact, the Rgg family was first named as *r*egulator *gene* of *g*lucosyltransferase, *gtfG,* in *S. gordonii* as a member of a family of streptococcal positive regulatory genes. ^14, 81^ It was demonstrated in this species that the glucosyltransferase gene, *gtfG*, was involved in the formation of glucan from sucrose, and regulated via a positive transcriptional regulatory determinant that the authors named *rgg,* as well as a putative protein designated as *rggD.* Both Rgg and RggD were found to have a similar helix-turn-helix domain at the N-terminus and 220 amino acids region rich in alpha helices at the C-terminal region, suggesting they belonged to the same family of transcriptional regulators. ^3, 82– 84^ Minimal work on these systems in *S. gordonii* outside of their impact on glucosyltransferases has been performed. Orthologs of these were later found in *S. pyogenes* and *S. thermophilus* to rely on SHPs to exert their activities, but *S. gordonii* was the first organism in which Rggs were defined as transcriptional regulators.

### Streptococcus mutans

The quorum sensing systems that have been best described in *S. mutans* are ones involved in competence development, ^3, 9, 10, 17, 85^ including the ComCDE and ComR systems. We discuss these later on in the review briefly. Rgg/SHP systems, while related to these quorum sensing systems, have not been studied extensively in this organism.

One Rgg/SHP quorum sensing system has been investigated in this organism, that regulates a specialized biosynthetic operon producing a RaS-RiPP . In *S. mutans* UA159, the Rgg/SHP in question encodes a SHP in a small open reading frame adjacent to the Rgg which was renamed PdrA (SMU_1509; Table 1) for **p**heromone **d**ependent **r**egulator of RiPP. The operon regulated by PdrA was found to regulate the RaS-RiPP named Tryglysin B (TryB). ^30^ Like other Rgg/SHP systems, induction of the RaS-RiPP relies on the presence of Rgg and SHP ^25, 30^ and requires the proteins PptAB for SHP import and OppD for SHP export. Tryglysins are the first founding members of a new subclass of RiPPs in *S. mutans* and the related species *S. ferus.* These peptides are ribosomally encoded and then modified to create a tetrahydro[5,6]benzindole motif. ^23^ These 7-mer peptides have bacteriostatic activity towards other streptococci with complete growth inhibition of *S. mitis, Streptococcus oralis, S. pneumoniae,* and *S. sanguinis* at 100 nM tryglysin treatment. *S. pyogenes, Lactococcus lactis,* and *Enterococcus faecalis* were unaffected under tryglysin exposure. However, the mechanism of tryglysin-mediated inhibition is unknown. ^30^

### Streptococcus ferus

While *S. ferus* is known to encode for several Rgg/SHP systems, including the tryglysin production system, ^23, 86^ little is known about how these systems function in this organism. Several proteins with similarity to Rgg and ComR regulators have been observed in *S. ferus* genomes via genome analysis. In the type strain of *S. ferus* (DSM 20646), this includes four ComR/Rgg like proteins: a canonical competence regulator *comR* (A3GY_RS0108865), a secondary ComR-like protein *comR2* (A3GY_RS0106270), *rggA* (A3GY_RS0105975), and *pdrA* (A3GY_RS0100490), which regulates the Rgg/SHP system involved in tryglysin biosynthesis. ^86^ ComR is the main competence regulator in *S. ferus*, relies on XIP induction, and behaves similarly to other ComR systems. ^86^ While it is known that *S. ferus* produces tryglysin A (TryA), if this Rgg/SHP system functions similarly to the *S. mutans* ortholog has not been thoroughly characterized. As such, much remains to be discovered concerning Rgg/SHP regulation in this species.

### Streptococcus thermophilus

*S. thermophilus* has been demonstrated to encode for multiple Rgg/SHP systems. ^37^ Rgg1358 ( Table 1) was the first Rgg demonstrated to act in quorum sensing in streptococci and to rely on a SHP for its induction. ^34, 87^ Rgg1358 relies on SHP1358 ( Table 1) for its activity, and controls the expression of another peptide called Pep1357c, later renamed as streptide. ^5, 87, 88^ Streptide was shown to rely on a radical SAM enzyme and an efflux transporter that matured and secreted the peptide outside of the cell. ^88^ This was actually the first demonstration of the existence of Rgg/SHP regulation of RaS-RiPPs, although at the time it was not realized how widespread these systems were. After its discovery, researchers also identified additional Rgg/SHP systems in *S. thermophilus.* This included Rgg1299/SHP1299 ( Table 1), which was demonstrated to function as a Rgg/SHP system, although the function of its gene targets is unknown, ^25, 89^ Rgg9420/SHP279 and Rgg7530/SHP273, although these identified SHPs were not expressed under experimental conditions. ^89^

Other Rgg systems, such as Rgg0182, ^90^ RggC, ^91^ and multiple newly identified Rgg systems that regulate RaS-RiPPs ^37^ are additionally encoded by various strains of *S. thermophilus.* These systems have either been directly demonstrated to rely on SHPs to exert their effects or are theorized to do so. ^89, 90^ *S. thermophilus* also encodes for a ComRS system, which induces competence via XIP. ^17^ Again, these proteins have various loci at which they target for regulation. ^89^ Rgg0812 regulates genes involved heat shock adaptation, ^90^ whereas RggC has been reported to impact oxidative stress response, but target genes have not been characterized. ^91^ Finally, several *S. thermophilus* strains (JIM8232, CNRZ1066) use Rgg/SHP systems to control the production of downstream RaS-RiPPs. These include RaS-RiPPs such as: streptide (SHP/Rgg _gp_sali_6_), streptosactins (SHP/Rgg _Sthermo_13_), bicyclostreptins (SHP/Rgg _gp_sali_4_), enteropeptins (SHP/Rgg _gp_sali_5_), and ryptides (SHP/Rgg _gp_sali_7_) ( Table 1). ^37^

Interestingly, Rgg/SHPs appear to be overrepresented in *S. thermophilus* compared to other streptococcal species. In a recent study, different strains of *S. thermophilus* were screened for Rggs and similar transcriptional regulators. It was found that *S. thermophilus* strains encode for a high density of Rgg or Rgg-like regulators. ^37^ Of the Rgg/SHP subfamily, half of these were found to be encoded next to ThiF or SAM radical enzymes (presumably RaS-RiPPs). ^37, 92^ These data indicate that Rggs and thus RaS-RiPPs appear to be overrepresented in this species. Therefore, the Rgg/SHP subfamily is widespread in *S. thermophilus* with functional systems regulating several biological activities.

### Other streptococci

Multiple other streptococcal species utilize the Rgg/SHP-type quorum sensing systems, suggesting a widespread role of this system in communication. This review cannot cover all defined systems present in streptococci, but we mention several additional species here.

Group B Streptococcus (GBS, otherwise known as *S. agalactiae*) species carry an Rgg2 ortholog called RovS and its associated small peptide SHP1520. This has been demonstrated to impact virulence and be regulated and produced in a similar manner to other Rgg/SHP systems. ^29^ This system performs inter-species crosstalk, with SHP1520 being able to activate QS in Group A Streptococcus. Targets of this system include the gene *gbs1556,* a transglutaminase/protease enzyme that is important GBS infection of macrophages, ^93– 95^ and *fbsA,* an adhesin. Disruption of *shp* and *rovS* genes result in moderate decrease in adherence of GBS and invasion of human HepG2 hepatic cells. ^29^ Overall, the RovS/SHP system regulates GBS virulence and contributes to bacterial pathogenesis. GBS has also been demonstrated to possess other Rgg systems as well. ^25^

Other streptococcal species for which Rgg/SHP QS has been described include *S. dysgalactiae* subsp. *equisimilis,* ^15^ *S. macedonicus, S. infantarius,* ^3, 15^ *S. porcinus,* ^15^ and *S. zooepidemicus* ^8^ ( Table 1).

### A brief overview of natural competence in streptococci

ComCDE and ComR are peptide-dependent quorum sensing regulators that induce competence in various streptococcal species; however, they operate quite differently from each other and are considered distinct from Rgg/SHP systems. ComX (also known as SigX), an alternative sigma factor, is critical for genetic transformation in streptococci as it drives the transcription of late-competence genes. ^47, 96^ Streptococci have been demonstrated to possess either ComCDE, ComR, or both of these systems, depending on the species. We discuss this briefly in a species that contains both of these systems: *S. mutans.*

**Figure 2.**
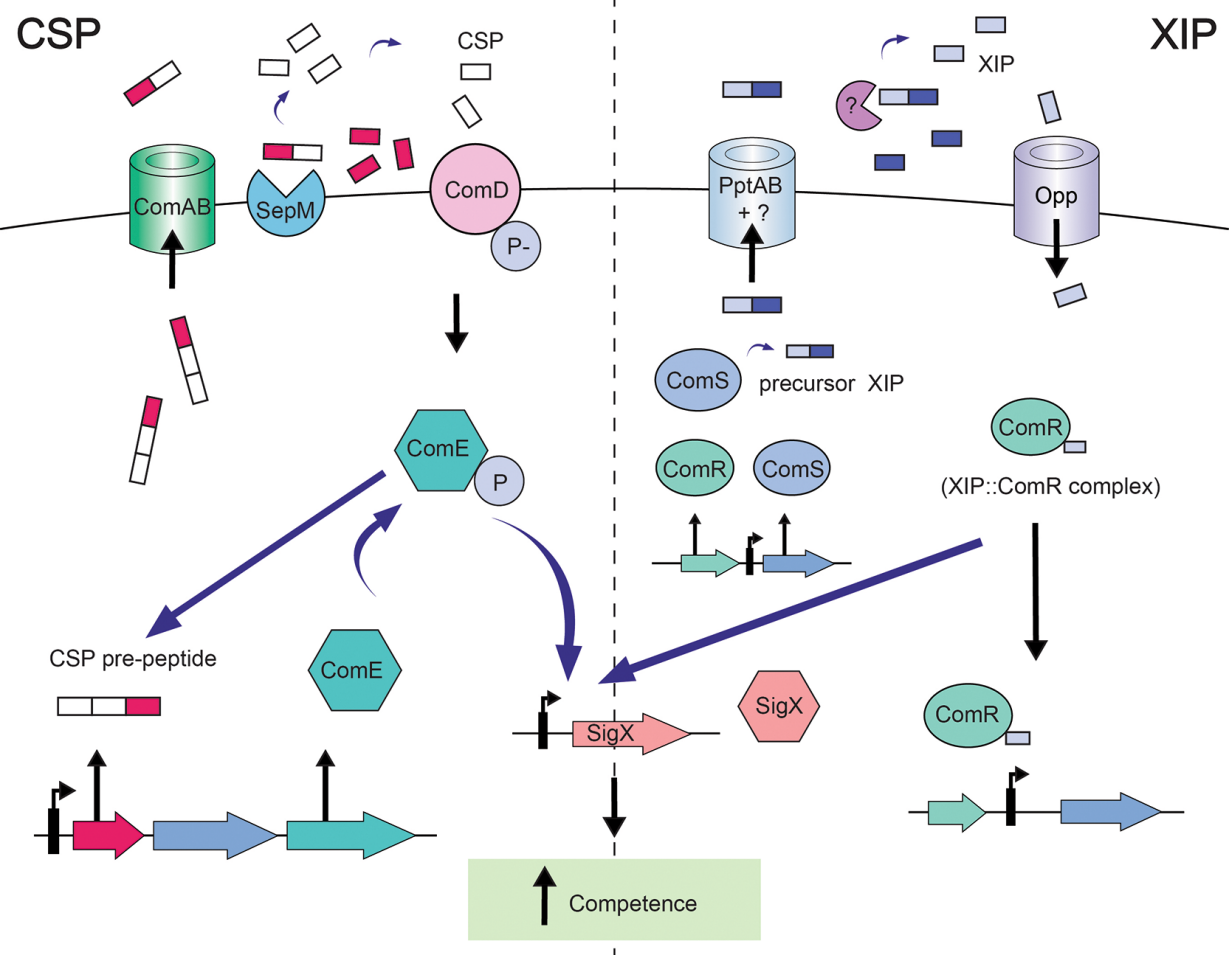
An overview of the competence mechanism of CSP and XIP in streptococci, using *S. mutans* as the prototypical example. In the CSP mechanism, ComC precursor CSP peptide is exported outside the cell through ComAB. Peptides are trimmed by SepM. ComD is phosphorylated, and phosphotransfer to ComE occurs. Phosphorylated ComE upregulates the *sigX* (also called *comX*) and leads to an increase in competence genes. In the XIP mechanism, ComS (XIP precursor) is exported through PptAB. ComS peptides are trimmed to form XIP and imported back into the cell through Opp. XIP binds to ComR, resulting in activation and upregulation of competence gene expression as a result of induction of the alternative sigma factor SigX/ComX.

In *S. mutans*, the activity of ComX is modulated by two signaling pathways, ComCDE and ComRS, that respond to competence stimulating peptide (CSP) and SigX-inducing peptide (XIP), respectively. ^97^ For the ComCDE system, competence is initiated via the binding of CSP to ComDE. ^98^ When CSP is secreted and at high density outside the cell, it can interact with ComD. Once sensed, ComD is autophosphorylated and the phosphorylation signal is transmitted to the cognate response regulator ComE ( Figure 2). ^98^ This activation induces the expression of *comX,* an alternative sigma factor, and as a result expression of competence related genes. ^85, 99^

The second way that competence induction can occur in streptococci is via the ComRS pathway. This is comprised of ComR and the XIP signaling peptide (encoded by *comS*). The XIP is 7 amino-acids long and is derived from the C-terminal region of a 17 amino-acid precursor ComS peptide. The precursor ComS peptide is translated, exported to the extracellular space, and cleaved by proteases to produce XIP. XIP peptide present in the extracellular space is then re-imported into the cell via the oligopeptide transporter Opp. When inside the cell, XIP binds to ComR regulator to drive the transcriptional expression of *comX* and *comS* promoters. XIP undergoes a positive feedback loop that amplifies the transcription of ComS, thereby producing increased levels of ComS/XIP ( Figure 2). ^9, 44, 100– 102^ ComR binding to XIP in turn induces the expression of *comX* and competence related genes. ^9^

As previously mentioned, how these systems are integrated is different depending on the streptococcal species. Some species, such as *S. mutans,* utilizes both ComCDE and ComRS type systems ( Table 1). ^9, 10, 44, 97, 102^ Other species contain only the ComCDE or ComRS system. For example, *S. ferus* exhibits a natural transformation system similar to *S. mutans,* but only possesses the ComRS system ( Table 1). ^86^ In contrast, *S. pneumoniae*, the best characterized organism in terms of competence, only contains the ComCDE system. ^103^ There are variations on the ComRS and ComCDE systems from their canonical classification, with different XIP or CSP motifs ( Table 1, for brevity only XIP sequences are listed) and expression profiles seen in species such as *S. thermophilus*, *S. suis, S. mitis, S. anginosus, and S. salivarius.* ^5, 17, 34, 100, 104, 105^ For reviews covering competence in various streptococcal species, we refer the readers to. ^106, 107^

### RaS-RiPPs as natural products and targets of Rgg/SHP QS

RaS-RiPPs have recently emerged as a large family of natural products regulated by Rgg/SHP systems via quorum sensing. RaS-RiPPs are ribosomally translated peptides that are post-translationally modified by RaS enzymes that install complex modifications. ^23^ First classified as a superfamily in 2001, Radical *S*-adenosylmethionine (RaS) enzymes have been of interest due to their ability to catalyze complex cellular reactions across all domains of life. ^108^ They are considered one of the most versatile biochemical enzyme superfamilies with over 100,000 orthologous enzymes. ^109^ The enzymatic function is initiated by a radical reaction in which cofactor SAM binds via its α-amino and carboxylate groups to a [4Fe–4S] ^+^ cluster in the RaS enzyme. This bond is reductively cleaved, typically leading to the production of 5′-deoxyadenosyl radical (5′-dA•). SAM enzymes thus function as cellular methylating agents and donate methyl groups to various acceptors such as DNA, proteins, and other small molecules. ^109^ The action of SAM methylation can lead to processes within the cell including gene regulation and the biosynthesis of metabolites. ^110– 112^

In streptococci, RaS metalloenzymes catalyze modifications on their respective precursor peptides during RiPP biosynthesis. This class of natural products detailed here are called RaS-RiPPs, a specific subtype of RiPPs that post-translationally modified by RaS-enzymes. ^23^ During RiPP biosynthesis, a precursor peptide composed of a N-terminal region (leader peptide) and a C-terminal region (core peptide) is synthesized by the ribosome, modified by tailoring enzymes, trimmed and modified to form the final natural product. ^43, 113^ A seminal study revealed Rggs are linked to RaS-RiPPs and found that streptococci possess 16 distant Rgg/RaS-RiPP subfamilies. These subfamilies are named based on the conserved motifs within their precursor peptide sequences. ^36^ RaS-RiPP subfamilies identified include the following: TQQ, WGK, str, GGG, KGR, HGH, CGx, SSH, KIS, RRR, GRC, QMP, NxxC, NEF, VSA, and CGG. The TQQ cluster is the largest subfamily and is primarily found in *S. suis.* Most RaS-RiPP subfamilies are produced by multiple streptococcal species ( Table 2). ^23^ It is thought that RaS-RiPP operons are typically controlled by their upstream Rgg/SHP quorum sensing systems. This has been experimentally demonstrated for *S. mutans* and *S. thermophilus* RaS-RiPP systems. ^30, 37^ There are only a few exceptions being CGx, CGG, and VSA in which the Rgg does not have a predicted SHP, although this could be due to lack of annotation of cognate SHPs. ^23^ Alternatively, these could represent Rggs regulated by LCPs. Typical organization of RaS-RiPP operons includes a precursor peptide for the RaS-RiPP, a RaS enzyme, and a transporter system. ^23, 114^ These systems can also encode for additional modifying enzymes, iron-sulfur proteins, ThiF-like proteins, and RiPP recognition elements. ^23^ As of 2026, many of these families in streptococci have been demonstrated to produce distinct peptides, ^115^ each possessing unique modifications that we discuss below.

**Table 2.** Experimentally established or predicted RaS-RiPPs and their functions.

Peptide	Producer Streptococci	Function	Reference
**Threoglucins/Rotapeptides (TQQ)**			
Threoglucins	*S. suis*	Growth inhibition of *S. suis*	^126^
Other threoglucins	*S. suis, S. suis sv., S. azizii*	Function not characterized	^23, 116^
**Tryglysins (WGK)**			
Tryglysin A	*S. ferus*	Growth inhibition of *S. ferus and* other streptococcal species	^30^
Tryglysin B	*S. mutans*	Growth inhibition of *S. mutans* and other streptococcal species	^30^
Other tryglysins	*S. equi zooepidemicus, S. equinus, S. ferus, S. mutans, S. sp.*	Function not characterized	^23^
**Streptides (KxxxW)**			
Streptide (also called Pep1357C)	*S. thermophilus*	Function not characterized	^88^
Other streptides	*S. agalactiae, S. mitis, S. suis, S. thermophilus*	Function not characterized	^23, 137^
**Streptosactins (GGG)**			
Streptosactin	*S. thermophilus*	Putative fratricidal agent	^114^
Other streptosactins	*S. constellatus pharyngis, S. gordonii, S. oralis oralis, S. oralis tigurinus, S. parasanguinis, S.* spp. *, S. thermophilus*	Function not characterized	^23^
**Enteropeptins (KGR)**			
Enteropeptins	*S. thermophilus*; *Enterococcus cecorum*	Growth inhibition of *E. cecorum* producer strain	^23, 129^
**Bicyclostreptins (HGH)**			
Bicyclostrepin A	*S. thermophilus*	Growth inhibition of *S. thermophilus* producer and other strains	^120^
Bicyclostrepin B	*S. thermophilus*	Function not characterized	^120^
Bicyclostrepin C	*S. agalactiae*	Growth inhibition of *S. thermophilus*	^120^
Other bicyclostrepins	*S. agalactiae, S. equi zooepidemicus, S. intermedius, S. mitis, S. thermophilus*	Function not characterized	^23^
**CGx**			
CGx	*S. equi ruminatorum, S. mitis, S.* spp. *, S. suis sv., S. thermophilus*	Function not characterized	^23^
**SSH**			
SSH	*S. equi zooepidemicus, S. mitis, S. parasanguinis, S.* spp.	Function not characterized	^23^
**KIS**			
KIS	*S. suis*	Function not characterized	^23^
**Ryptides (RRR)**			
Ryptides	*S. parauberis, S. suis*	Function not characterized	^23, 119^
**GRC**			
GRC	*S. pneumoniae, S. oralis*	Function not characterized	^23, 115^
**QMP**			
Suisactin	*S. suis*	Function not characterized	^23, 118^
**NxxC**			
NxxC	*S. orisratti*, *S. porci, S. equi zooepidemicus*	Function not characterized	^23, 117^
**NEF**			
NEF	*S. mitis, S. marmotae*	Function not characterized	^23^
**VSA**			
VSA	*S.* spp.	Function not characterized	^23^
**CGG**			
CGG	*S. orisratti*	Function not characterized	^23^

### Chemistry defines RaS-RiPPs

**Figure 3.**
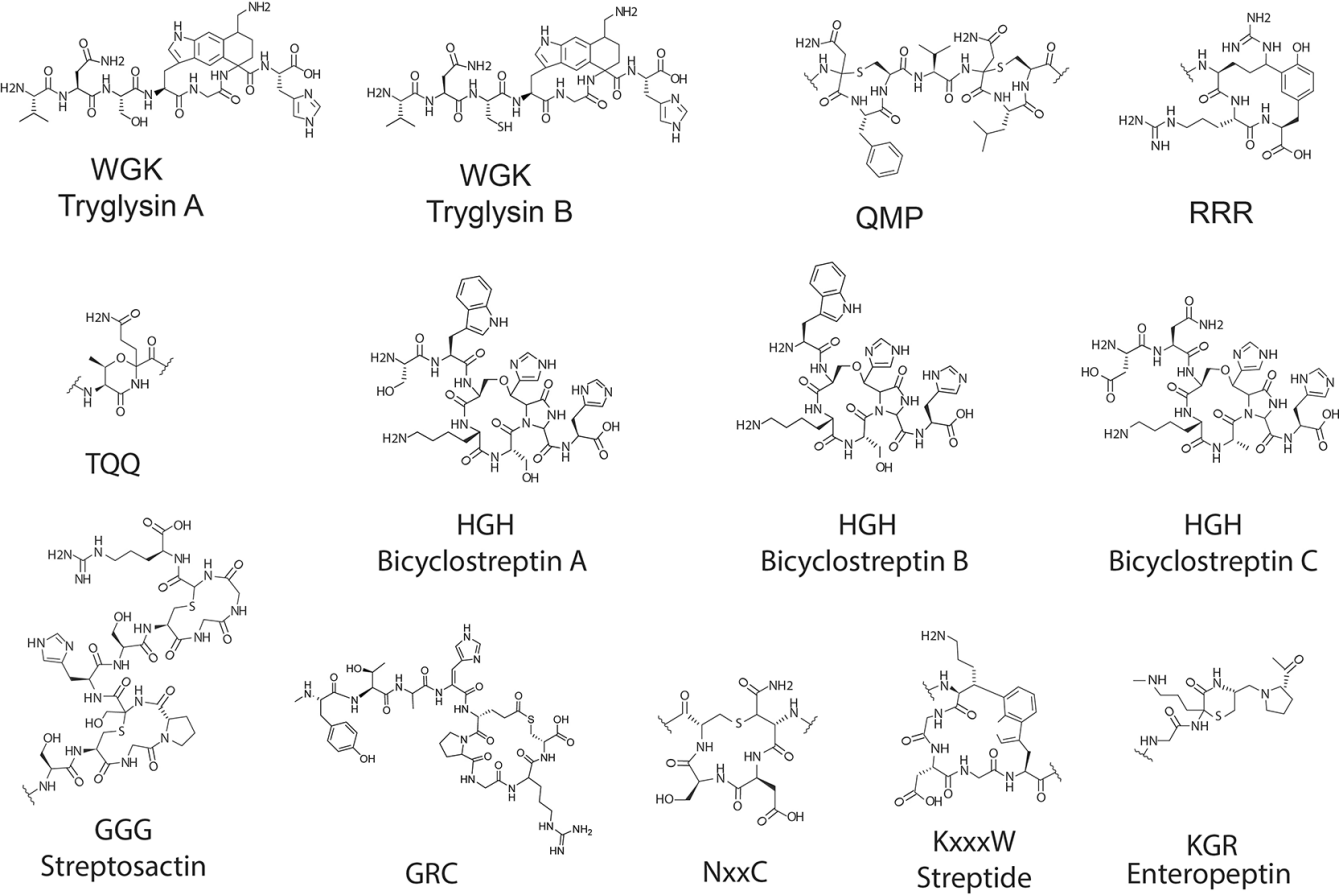
RaS-RiPP structures currently identified or predicted from streptococcal RaS-RiPP subfamilies with the exception of Enteropeptin from *Enterococcus cecorum.* Subfamilies for which structures are shown include WGK (Tryglysin A and B), QMP, RRR (Ryptides), TQQ (Threoglucins/Rotapeptides), HGH (Bicyclostreptin A, B, and C), GGG (Streptosactin), GRC, NxxC, KxxW (Streptide), and KGR (Enteropeptin).

RaS enzymes catalyze various modifications creating unique motifs across numerous superfamilies of RaS-RiPPs. ^35, 115^ Modifications that have been found to present in RaS-RiPPs include heterocycles formed by linkages between Lys-Trp residues, β-thioether linkages, and sactionine bridges. For instance, TqqB, the RaS enzyme from the TQQ subfamily, demonstrated the first observable ether modification from these systems. TqqB catalyzes the formation of the ether cross-link through joining the threonine side chain oxygen to the α-carbon of the adjacent glutamine residue in TqqA, forming threoglucin ( Figure 3). ^116^ We briefly discuss other documented modifications discovered below.

Within the NxxC subfamily, RaS enzyme NxxB installs an intramolecular β-thioether bond onto its substrate peptide though the connection of Cys-thiol to the β-carbon of an upstream Asn residue ( Figure 3). ^117^ Biosynthetic gene clusters for tryglysin ( *wgk*) and streptide (also called KxxxW, *str*, *aga*, and *sui*) encode for RaS enzymes that introduce Lys-Trp linkages ( Figure 3). ^23, 36^ Streptide was the first demonstration of an Rgg-linked RaS-RiPP, although at the time it was not realized how broad this distribution across streptococci truly was. ^5^

Sactionine bridges have been observed in the GGG and QMP subfamilies, with resulting RaS-RiPPs having two sactionine bridges present in their structures ( Figure 3). ^114, 118^ The QMP subfamily yields suisactin, whereas streptococci that possesses the GGG subfamily produces streptosactin. Both of these yield unique peptides and rely on the enzymes encoded for in the RaS-RiPP operon to produce the end products. ^114, 118^ Other modifications have been observed in RaS-RiPP families, such as an arginine-tyrosine crosslink in peptide structures within the RRR (also called ryptides) subfamily. ^119^ In the HGH subfamily, numerous forms of peptides are produced known as bicyclostreptins in which a macrocyclic beta-ether and heterocyclic linkages between backbone amide nitrogen and adjacent alpha-carbon are formed ( Figure 3). ^120^ GRC peptides form a C-terminal Glu-to-Cys thiolactone macrocycle and generates L- *allo*-Thr and didehydrohistidine. ^37, 115^ In the KGR subfamily, there are currently no known structures from streptococcus; however, structures have been defined from enterococcus termed enteropeptins, which are small sactipeptides containing a thiomorpholine ring ( Figure 3). ^121^ Characterized structures of mature RaS-RiPP products have been elucidated through the work of the Seyedsayamdost laboratory ( Figure 3).

### RaS-RiPPs with antimicrobial properties

Some subfamilies of RaS-RiPPs have been found to possess inhibitory properties ( Table 2). Members of the WGK RaS-RiPP subfamily, Tryglysin A and Tryglysin B produced by *S. ferus* and predicted in *S. mutans,* respectively, have bacteriostatic activity towards other streptococci. ^30^ *S. mutans* is a streptococcal species that results in cavities in humans, while *S. ferus* was isolated from the oral cavity of rats and later isolated from pigs. ^122, 123^ Tryglysins (TryA and TryB) inhibit the growth of other streptococci such as *S. oralis*, *S. sanguinis*, *S. pneumoniae* at 100 nM concentrations. ^30^ Due to *S. mutans* and *S. ferus’* involvement in the oral cavity, it stands to reason that tryglysin could be used by these species to interact with oral communities. A recent study took the first steps in defining the impact of TryA on *ex-vivo* oral microbiomes. It was found that a saliva derived oral inoculum had delayed growth and acidification in a chemically defined media (CDM) upon addition of tryglysin compared to control conditions. Shotgun metagenomics revealed that growth in CDM resulted in the streptococcal species *S. salivarius* dominating the culture under anaerobic conditions. Tryglysin addition was marked by a concomitant increase of *Saccharibacteria.* ^38^ However, due to the inactivity of tryglysin under typical saliva culturing conditions, findings were limited. Further testing within a media that can support a wide variety of oral species and tryglysin activity will be needed to understand oral cavity interactions.

Other research in streptococci has been focused on the sactipeptide termed streptosactin (GGG subfamily). Streptosactin, consisting of a 14-mer peptide with a pair of 4-residue sactionine macrocycles, inhibits growth of the producing host, *S. thermophilus* with 1 μM of streptosactin causing complete growth inhibition. Streptosactin biosynthesis is correlated with the expression of early competence genes, and as such it has been proposed that streptosactin is the first fratricidal agent in *S. thermophilus.* This is primarily due to its ability to effectively exhibit self-killing activity as well as an observable cell-clumping when streptosactin is present. ^114, 124, 125^

Threoglucins (also called rotapeptides) are a novel 1,3-oxazinane heterocycle-containing family of peptides that belong to the TQQ subfamily. Threoglucins are inhibitory towards their producer species *S. suis* at 500 nM. They do not appear to impact the growth of other streptococcal species, ^126^ but modulate the sensitivity of *S. suis* to other antibiotics. For instance, simultaneous application of 2 μM threoglucins A/B with 200 μM ciprofloxacin resulted in significantly higher viability than 200 μM ciprofloxacin alone. This reveals the potential for threoglucins to serve as a growth-curbing signal while allowing *S. suis* to increase tolerance towards toxins or antibiotics. ^126^

Bicyclostreptins (HGH subfamily) are another class of RaS-RiPPs for which bacteriostatic activity has been observed. These have been isolated from culture supernatants of probiotic *S. thermophilus* as well as *S. agalactiae* at nanomolar concentrations. Several variants of bicyclostreptins have been documented, with Bicyclostreptin A and B isolated from *S. thermophilus,* ^127^ and Bicyclostreptin C was isolated from *S. agalactiae.* ^128^ Bicyclostreptins have bacteriostatic activity against some *S. thermophilus* strains, as well as their producing hosts. Activity of Bicyclostreptin C can be overcome by producer species, as application does not result in a permanent growth inhibition, suggesting that this peptide is degraded, resistant strains can emerge, or subpopulations of immune producer cells can arise. ^120^

Finally, enteropeptins (KGR subfamily), while only being characterized from *Enterococcus cecorum*, also have narrow-spectrum bacteriostatic activity. Enteropeptin A specifically inhibits the growth of *E. cecorum*, but not other bacterial species such as *S. thermophilus or E. faecalis.* At physiological production levels (1 μM) *E. cecorum* could recover from enteropeptin inhibition, but higher concentrations were almost completely inhibitory at least out to 18 hours of growth. Again, the mechanism of inhibition is unknown, and the exact reasons for production unclear. ^129^

Further research on growth inhibition mechanisms and interactions with bacterial species of the aforementioned RaS-RiPPs and other unexplored families is needed to establish their role in cell physiology and mechanisms of action.

### RaS-RiPPs’ and Rgg/SHP interplay

**Figure 4.**
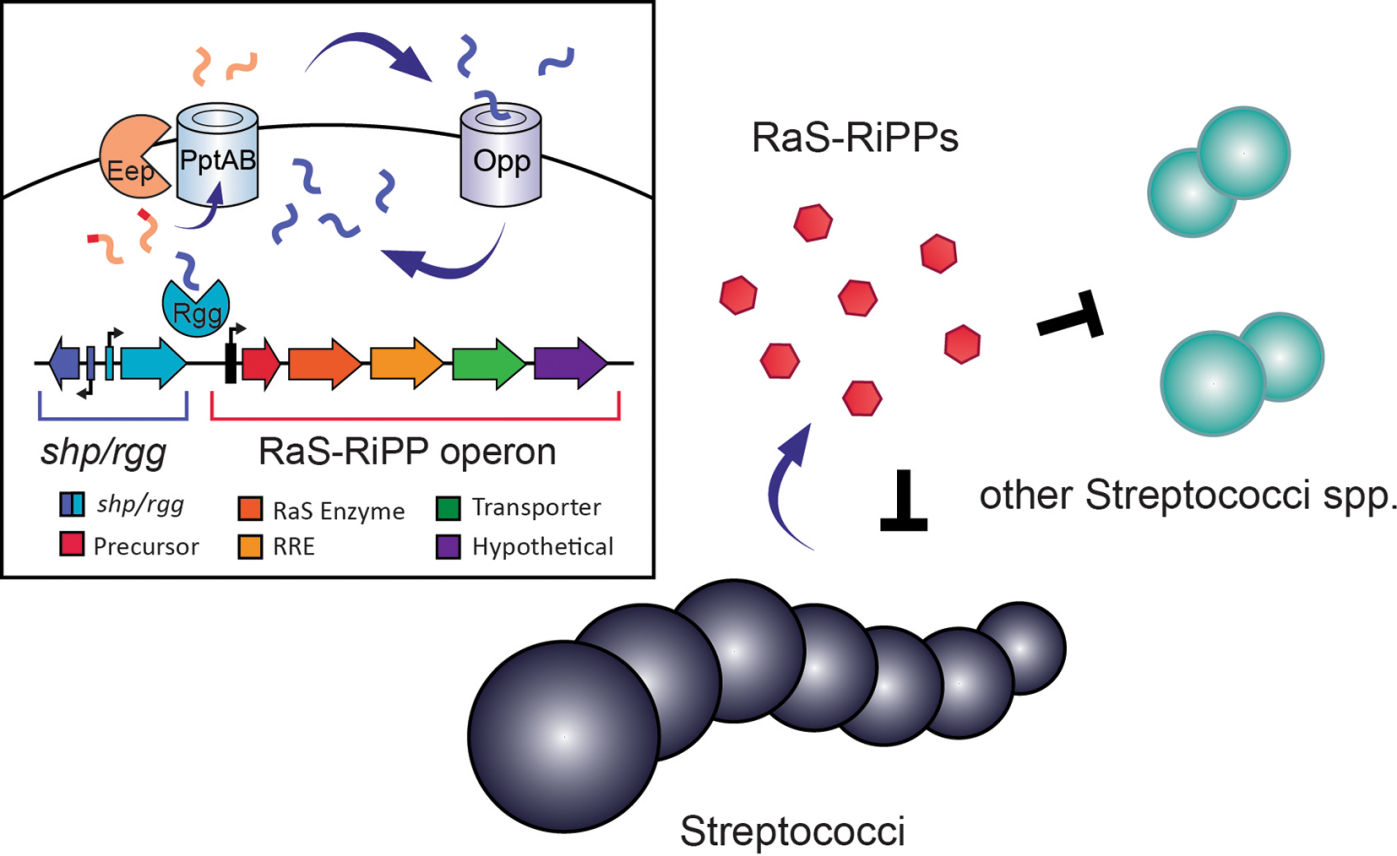
Rgg/SHP quorum sensing in streptococci and RaS-RiPP induction. SHP precursor peptides are trimmed by Eep or alternative peptidases and transported into the extracellular matrix through PptAB. Modification to the peptide is known to occur in some cases in the extracellular matrix. The mature peptide is transported back into the cytoplasm through the Opp transporter where it can bind to Rgg. The Rgg/SHP complex binds to the RaS-RiPP operon and drives the expression of RaS-RiPP natural products. A RaS-RiPP operon can consist of genes including (from left to right) a peptide precursor, RaS enzyme, RiPP Recognition Element (RRE), transporter, and occasionally hypothetical genes. Components of operons can vary between subfamilies, with some lacking certain genes, having two RaS enzymes. RaS-RiPP natural products can inhibit growth of other streptococci, the producing species, or serve as growth regulatory signals.

As previously described, Rgg/SHP quorum sensing systems play an important role in the production of RaS-RiPPs. Rgg/SHP operons are specific to streptococci and can regulate expression of virulence genes as well as a host of other processes. ^3, 5, 130^ Rgg/SHP systems also have been found to regulate the production of RaS-RiPP natural products, one example being streptides. Streptides’ production are driven by an Rgg/SHP system that is triggered by high cell density ^117^ and their discovery was the first demonstration of induction of a RaS-RiPP by Rgg/SHP QS. Another system that has been shown to be Rgg/SHP QS dependent is the tryglysin operon from the species *S. mutans.* ^30^ Further supporting this link between Rgg transcriptional regulators and RaS-RiPPs, a recent study demonstrated that bicyclostreptins also appear to be modulated by their cognate Rgg/SHP systems in *S. thermophilus* JIM8232. ^37^ Rgg/SHP operons are commonly found upstream of RaS-RiPP operons in streptococci, as previously mentioned. In 2018, a seminal study found that in a bioinformatic search of 2875 streptococcal genomes for RaS enzyme encoding genes and Rgg encoding genes within a 1–3 gene distance, 592 RaS-RiPP gene clusters were identified that were predicted to be controlled by an Rgg/SHP quorum sensing locus. These gene clusters were further separated into the 16 RaS-RiPP subfamilies. Each subfamily is predicted to be controlled by a Rgg/SHP system with the exception of CGx, CGG, and VSA in which the divergently transcribed *shp* was not identified despite having an associated *rgg.* ^23^ Production of these natural products has been shown to correlate with cell density, providing further evidence for Rgg/SHP regulation of these systems. When a high cell density is present, SHPs are imported into the cell which bind to the Rgg transcriptional regulator leading to the expression of the RaS-RiPP operon ( Figure 4). For example, streptosactin and bicyclostreptin production in *S. thermophilus* are cell density dependent, as is Tryglysin A from *S. ferus*, and TQQ from *S. suis.* ^30, 37, 114, 116, 120^

As such, the use of Rgg/SHP systems control RaS-RiPP systems in streptococci appear to be a conserved mechanism used throughout the genus. While Rgg/SHP systems control other genes involved in virulence and colonization, the function of RaS-RiPPs appear to revolve around inter-bacterial competition and response to the environment. It stands to reason that Rgg transcriptional regulators have evolved to be pervasive throughout streptococci and have been co-opted to regulate many of their processes that are necessary for environmental survival, RaS-RiPP production being one of them. ^30, 114^

## Conclusion

Streptococci produce an array of small peptides that underly complex reactions in the cell. Although some streptococcal systems are vastly understudied, quorum sensing systems in general have been researched due to their significance to cellular processes. We discuss Rgg/SHP quorum sensing systems which are conserved throughout streptococcal species. These systems can control cellular colonization, virulence, biofilm formation, and even important metabolic programs. ^1, 3– 11^ Importantly, Rgg/SHP quorum sensing systems also regulate the production of streptococcal RaS-RiPPs. Streptococcal RaS-RiPPs are novel products that appear to have multifactorial effects, including inhibiting the growth of other streptococcal species, narrow spectrum activity towards strains of the producer species implying fratricidal effects, and impacts on antibiotic susceptibility. As such, these peptides prove of interest for further studies in terms of their mechanism and impact on cellular processes. The discovery of antibacterial activity of RaS-RiPPs such as tryglysins, streptosactins, enteropeptides, threoglucins and bicyclostreptins ^30, 114, 120, 126, 131^ also implies their importance for interbacterial competition in communities. With the presence of biosynthetic gene clusters in multiple species within most of the 16 subfamilies, ^23^ it presents the possibility that additional unidentified RaS-RiPP products might exist. With much more to uncover regarding streptococcal small peptides and RaS-RiPP mature products, this review presents an in-depth summary of our current knowledge today and provides insight for future research.

## Data Availability

No data is associated with this article.
