# Rgg/SHP transcriptional regulators, RaS-RiPPs, and their impacts in streptococci

**DOI:** 10.12688/f1000research.178446.2

**Published:** 2026-06-03

**Authors:** Sristi Dey, Sophie A. Krivograd, Britta E. Rued

**Affiliations:** 1 Veterinary Microbiology and Preventive Medicine, Iowa State University College of Veterinary Medicine, Ames, Iowa, 50010, USA

**Keywords:** Streptococci, Rgg/SHP, competence, quorum sensing, RaS-RiPPs

## Abstract

Streptococci are prevalent in animal and human microbiomes. These organisms produce a vast array of small peptides that modulate complex functions within the cell such as quorum sensing, virulence, and metabolism. Transcriptional regulators are central to this process, of which Rgg transcriptional regulators hold prominence in streptococci. These systems are controlled by peptides known as SHPs (short hydrophobic peptides) and LCPs (leaderless communication peptides). Also known as Rgg/SHP quorum sensing (QS) systems, they are ubiquitous across streptococcal species and regulate cellular competence, metabolic programs, virulence, and facilitate colonization of host species. It has been recently demonstrated that Rgg/SHP QS systems can also regulate the production of natural products known as RaS-RiPPs ( **Ra**dical ***S***-adenosylmethionine enzyme **Ri**bosomally translated and **P**ost-translationally modified **P**eptides). RaS-RiPPs are widespread in streptococci with sixteen current subfamilies. Some of these natural products possess inhibitory properties while others’ functions are currently unknown. We provide here a review of Rgg/SHP systems within streptococci, the complexities and characterized functions of RaS-RiPPs, as well as the connection between Rgg/SHP and RaS-RiPPs. We also provide a brief overview of competence in streptococci, given the relevance of these systems to peptide signaling in this genus.

## Introduction

**Figure 1.**
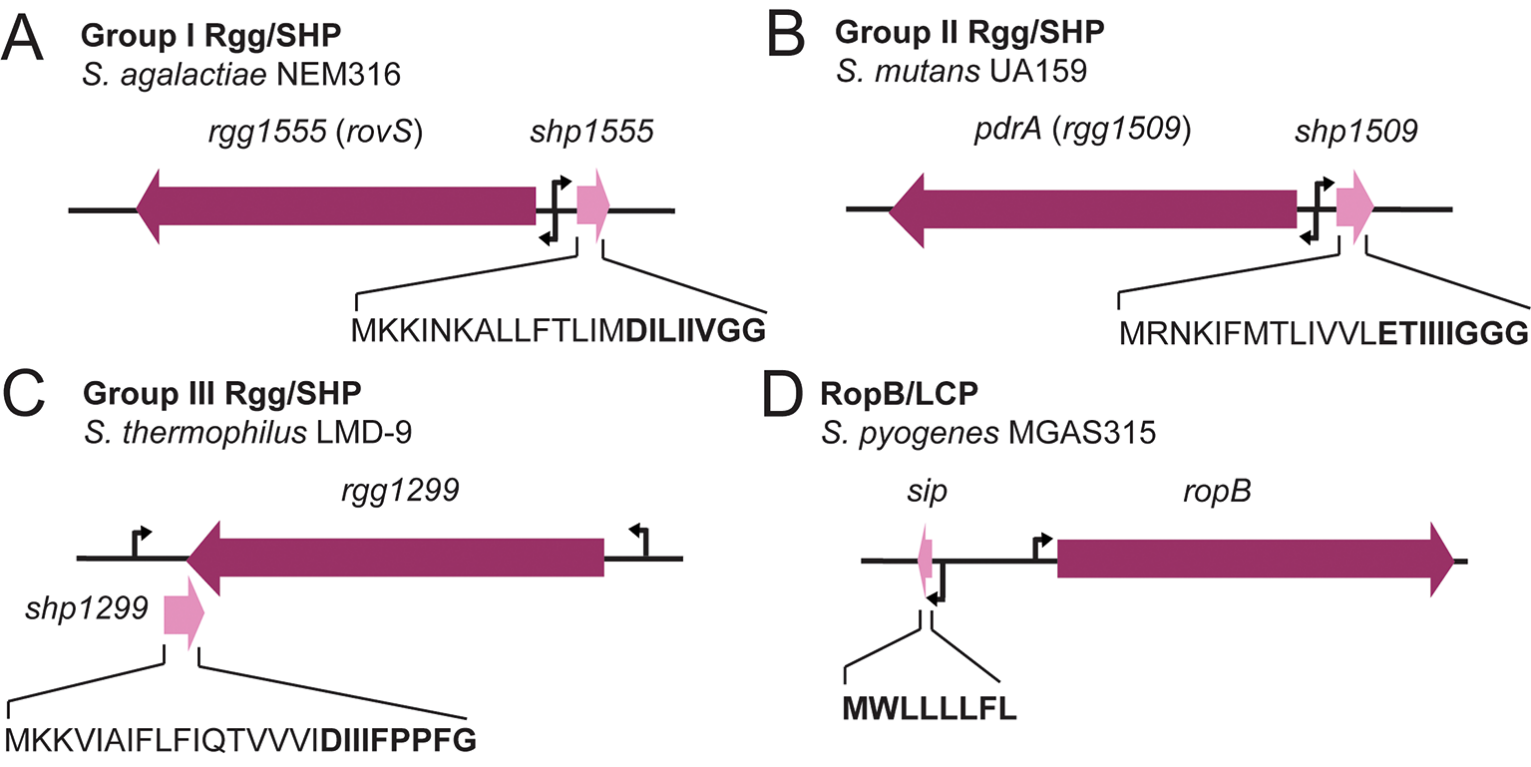
Classification of Rgg/SHP groups and LCP systems. Summary of Rgg/SHP group classification as defined by Fleuchot et al., ^5^ with representative systems being shown for each group. A) For Group I, *rgg* and *shp* genes are divergently transcribed, and SHPs contain a conserved N-terminal aspartate residue. The *rovS* locus from *S. agalactiae* NEM316 is shown as an example. B) For Group II, *rgg* and *shp* genes are also divergently transcribed, and SHPs contain a conserved N-terminal glutamate residue. The *pdrA* ( *rgg1509*) locus from *S. mutans* UA159 is shown an example. C) For Group III, SHPs overlap with the end of the *rgg* genes and are rich in hydrophobic amino acids. The *rgg1299* locus from *S. thermophilus* LMD-9 is shown as an example. D) For LCPs, these signaling peptides are generally divergently transcribed from their cognate Rggs (re-named RopB), and encode for short leaderless peptides, typically 8 to 10 amino acids long. The *ropB* locus from *S. pyogenes* MGAS315 is shown as an example.

Bacterial species respond to different stimuli essential for survival through cell density-linked signaling circuits called quorum sensing (QS) systems. These systems synchronously control gene expression through the production, release and detection of chemical signaling molecules known as “autoinducers” or “pheromones”. In response to cell density, these signals accumulate in the environment and are detected by cognate receptors to control cell-cell communication. ^1^ ^,^ ^2^ These systems have been demonstrated to control cellular processes involved in colonization, virulence, biofilm formation, and important metabolic programs. ^1^ ^,^ ^3^ ^–^ ^11^ In Gram-positive bacteria, QS mediated by peptide signals can function through two major mechanisms: **t**wo- **c**omponent **s**ystems (TCS) and control by members of the RRNPP superfamily of proteins. TCS are widespread in bacteria and control diverse cellular processes. These systems are typically comprised of a membrane-bound histidine kinase receptor and a cytoplasmic response regulator. In TCS, autoinducers are sensed by the histidine kinase receptor, leading to autophosphorylation of the kinase and then transferring this phosphate to an internal response regulator. The response regulator then modulates gene expression, resulting in changes in cellular behavior. ^12^ ^–^ ^14^ A classic example of this system in streptococci is ComCDE, which we discuss later. In contrast, members of the RRNPP family, standing for Rap, **r**esponse regulator **a**spartate **p**hosphatase; Rgg, **r**egulator **g**ene of **g**lucosyltransferase; NprR, **n**eutral **pr**otease **r**egulator; PlcR, **p**hospho **l**ipase **C r**egulator; and PrgX, **p**heromone- **r**esponsive **g**ene **X,** ^15^ ^,^ ^16^ have a different mechanism by which they sense autoinducers. Members of this superfamily are united by a conserved C-terminal tetracopeptide repeat (TPR) domain architecture, consisting of 6-9 TPR motifs or TPR-like domains that mediate peptide binding and typically respond to a small peptide autoinducer. This includes members that function as transcriptional regulators, and others such as Rap proteins that act as phosphatases or proteins that interact with response regulators to modulate their activity. ^17^ Those that are transcriptional regulators additionally possess a helix-turn-helix (HTH) domain at the N-terminus, allowing them to bind to regulatory targets. ^16^ ^,^ ^18^ Among these, Rgg transcriptional regulators represent a major class of proteins that control quorum sensing systems in streptococci. ^2^ ^,^ ^15^ ^,^ ^16^ ^,^ ^19^ ^,^ ^20^ These proteins interact with cognate peptide pheromones via TPR-like domains and bind to promoters that they regulate using a HTH domain. The peptide pheromones that the TPR like domain interacts with are called SHPs (short hydrophobic peptides) and as more recently demonstrated LCPs (leaderless communication peptides). ^7^ ^,^ ^8^ ^,^ ^11^ ^,^ ^19^ ^,^ ^21^ ^–^ ^23^ SHPs are often encoded directly next to genes that produce the Rgg transcriptional regulator ^2^ ^,^ ^3^ ^,^ ^5^ ^,^ ^7^ ^,^ ^24^ ^–^ ^27^ and contain an abundance of hydrophobic amino acids such as leucine, isoleucine, valine, and glycine. ^28^ ^,^ ^29^ The binding of a SHP peptide to its cognate Rgg results in a conformational shift, ^23^ and thus regulation of promoters at which the Rgg binds. This can result in transcriptional activation or repression, depending on the Rgg. ^3^ SHP peptides are essential for triggering this process and their sequence is Rgg system specific. ^3^ ^,^ ^7^ ^,^ ^30^ ^–^ ^33^ Classifications for these Rgg/SHP systems have been proposed based on the transcriptional orientation of SHPs and Rggs and the amino acid content of SHP peptides. ^5^ In this classification, Group I and II include SHPs that are divergently transcribed from the cognate Rgg regulator ( Figure 1A-B) and the SHP peptide contains an N-terminal conserved aspartate or glutamate. In contrast, Group III Rgg/SHP systems possess SHPs that overlap with their cognate Rgg gene at which they are convergently transcribed from ( Figure 1C). ^5^ ^,^ ^28^ However, as previously mentioned, it was recently discovered that there is another family of short peptides that bind to Rgg regulators in streptococci. These do not fit into the classification of Rgg/SHP systems, as they have characteristics that make them distinct. This subset is composed of leaderless peptides, generally 8 to 10 amino acids long, encoding for a mature signaling sequence without the leader sequences present in SHPs that are necessary for processing and secretion ( Figure 1D). These have been named LCPs and are widespread across streptococci and Firmicutes. ^34^ The first LCP to be discovered was found in *S. pyogenes*, in which SIP (SpeB-inducing peptide) activates the Rgg regulator also known as RopB. ^11^ Similar to SHPs, SIP and other LCPs are divergently transcribed from their Rgg regulator, typically named RopB ^11^ ^,^ ^34^ ( Figure 1D). As such, peptides involved in streptococcal QS can be split into SHPs and LCPs.

**Figure 2.**
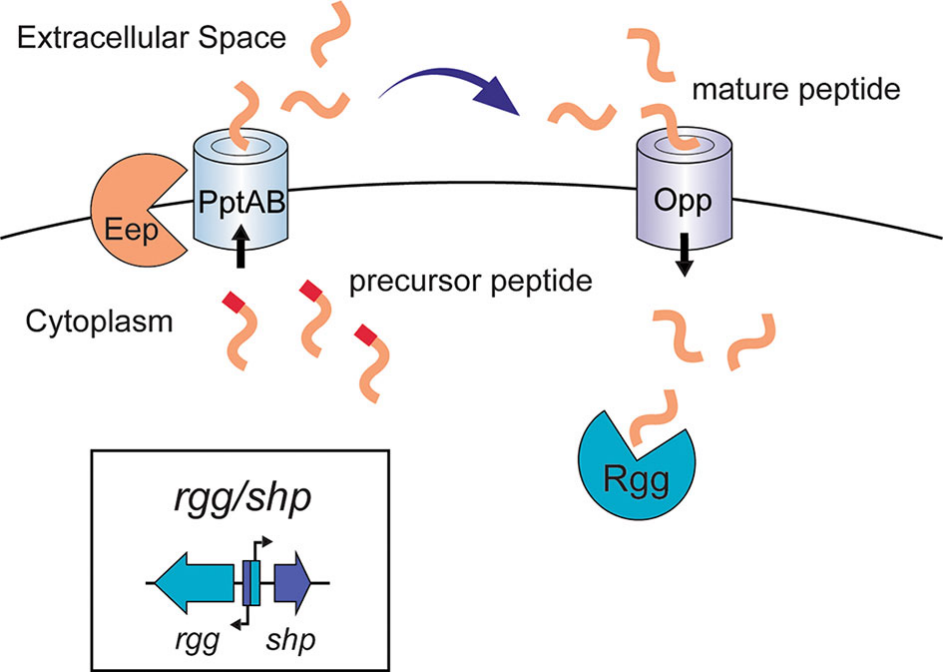
Overview of Rgg/SHP quorum sensing. Precursor peptides are trimmed by Eep or alternative peptidases. They require transport through PptAB to the extracellular space. Maturation of the SHP is known to occur in some cases in the extracellular matrix or during transport. The mature peptide is transported back into the cytoplasm through the Opp transporter where it can bind to Rgg, which induces downstream genes and increases production of the cognate SHP. An inset box depicts typical organization of Group I and Group II Rgg/SHP systems, where the promoters for *rgg* and *shp* genes are in the same intergenic region, located on opposite strands. One promoter drives *rgg* expression, and the other drives *shp* expression. This allows for simultaneous regulation of this region by a Rgg transcriptional regulator.

Orthologs of Rgg proteins are widespread in low G+C Gram-positive bacteria, ^5^ ^,^ ^16^ but Rgg/SHP regulators are concentrated in streptococcal species. Multiple studies have demonstrated the importance of these systems and related ComRS regulatory circuits in streptococci such as *S. pneumoniae, S. mutans*, *S. pyogenes*, *S. thermophilus, S. agalactiae,* and others. ^5^ ^,^ ^6^ ^,^ ^9^ ^,^ ^35^ ^–^ ^39^ A simplified example of Rgg/SHP (and LCP) signaling is shown in Figure 2, in which Rgg/SHP systems regulate target loci depending on their interaction with SHP peptides. These peptides are produced, processed and secreted, and then imported into cells as a mature form that binds to the cognate Rgg regulator, allowing the Rgg to regulate gene expression at the promoter level. The transcriptional regulation of these systems is species-specific and Rgg/SHP systems target unique loci depending on the streptococcal organisms they are present in. For example, these regulators play roles in colonization and host adaption in *S. pneumoniae,* ^2^ biofilm development, resistance to lysozyme, and immunosuppression in *S. pyogenes,* ^40^ ^–^ ^42^ and virulence factor regulation in *S. agalactiae*, ^32^ among other functions. Rgg/SHP systems have also been demonstrated to regulate operons that produce RaS-RiPPs ( **Ra**dical ***S***-adenosylmethionine enzyme and **Ri**bosomally translated and **P**ost translationally modified **P**eptides). ^18^ ^,^ ^26^ ^,^ ^33^ ^,^ ^43^ ^,^ ^44^ These systems encode small ribosomally translated peptides that are post-translationally modified by a radical *S*-adenosylmethionine (SAM) enzyme ^26^ ^,^ ^43^ and are typically secreted into the extracellular space. Some classes of RaS-RiPPs have been shown to inhibit other streptococcal species and have varying effects on antibiotic resistance and growth. ^33^ ^,^ ^45^ ^–^ ^49^ As such, in the past eight years these have begun to emerge as a suite of biosynthetic gene operons that are controlled by Rgg/SHP systems. This review aims to provide an overview of Rgg/SHP systems among streptococcal species, their connection to RaS-RiPP systems, as well as their effects on streptococcal physiology themselves.

Our review begins with discussion of the first streptococcal species in which Rgg/SHP systems were found to be QS regulatory circuits: *S. thermophilus* and *S. pyogenes*, followed by *S. pneumoniae* where these systems have also been extensively studied. We then introduce *S. gordonii*, in which Rgg transcriptional regulators were first observed, followed by discussion of Rgg systems in other streptococci, such as *S. mutans*, *S. ferus*, *S. agalactiae* and other relevant species. We then briefly discuss peptide signaling systems involved in competence, touching on ComCDE systems and ComR type regulators, given the vast amount of literature examining these QS systems in streptococci and ComR systems similarity to Rgg/SHP systems. This is followed by a discussion of RaS-RiPPs, their connection to Rgg/SHP QS, and their known functions.

### Streptococcus thermophilus

*S. thermophilus* was the first organism demonstrated to use Rgg/SHP systems for quorum sensing and has been demonstrated to encode for multiple Rgg/SHP systems. ^5^ ^,^ ^50^ Specifically, Rgg1358 ( Table 1) was the first Rgg demonstrated to be involved in quorum sensing in streptococci and to rely on a SHP for its induction. ^5^ ^,^ ^50^ Rgg1358 relies on SHP1358 ( Table 1) for its activity and controls the expression of an operon involved in the production of another peptide called Pep1357c, later renamed as streptide. ^50^ ^,^ ^51^ Streptide production requires a radical SAM enzyme (StrB) to generate a mature 9-mer product from a precursor peptide, and an efflux transporter to secrete the peptide outside of the cell. The exact mechanism of streptide processing during secretion is still unknown. ^51^ This was actually the first demonstration of the existence of Rgg/SHP regulation of RaS-RiPPs, although at the time it was not realized how widespread these systems were. After its discovery, researchers also identified additional Rgg/SHP systems in *S. thermophilus*. This included Rgg1299/SHP1299 ( Table 1), which was demonstrated to function as a Rgg/SHP system, although the function of its gene targets is unknown. ^28^ ^,^ ^52^ Rgg9420/SHP279 and Rgg7530/SHP273 were other systems that were reported, however, only *shp273* was not expressed under the experimental conditions tested, whereas *shp279* was detected at low levels. Notably, this study also further demonstrated that SHPs are secreted and subsequently reimported, which is consistent with the role of intracellular quorum sensing regulation. ^52^

**Table 1.** Experimentally confirmed SHP, LCP and XIP peptides.

Transcrip. Reg. ^**a**^	SHP/XIP sequence	Peptide name ^**b**^	Genome	Ref. ^**c**^
***S. thermophilus***				
Rgg1358	EGIIVIVVG	SHP1358/SHP768	*S. thermophilus* LMD-9	^28^ ^,^ ^52^ ^,^ ^56^
Rgg1299	DIIIFPPFG	SHP1299/SHP714	*S. thermophilus* LMD-9	^28^ ^,^ ^52^
Rgg9420	EGIIVIGVG	SHP279	*S. thermophilus* LMD-9	^52^
Rgg _Sthermo_6_	DIIIFPPFG	SHP _Sthermo_6_	*S. thermophilus* ST13, JIM8232	^18^
Rgg _Sthermo_12_	DIIIIVGG	SHP _Sthermo_12_	*S. thermophilus* STH_CIRM_1047, 252, CNRZ1066	
Rgg _Sthermo_13_	EGIIVIGVG	SHP _Sthermo_13_	*S. thermophilus* TSGB 4234, JIM8232	
Rgg _gp_sali_3_	CIYTIVGGV	SHP _gp_sali_3_	*S. thermophilus* STH_CIRM_1125, CNRZ1066, ena-SAMPLE-787-33427	
Rgg _gp_sali_4_	EIIIIIAL	SHP _gp_sali_4_	*S. thermophilus* STH_CIRM_1121, MV-FAST4, JIM8232	
Rgg _gp_sali_5_	ESIIVIAVG	SHP _gp_sali_5_	*S. thermophilus* ACA-DC 2, JIM8232, CNRZ1066	
Rgg _gp_sali_6_	EGIIVIVVG	SHP _gp_sali_6_	*S. thermophilus* St-10, Vach60, JIM8232	
ComR	IAILPYFAGCL	XIP (SHP0316)	*S. thermophilus* LMD-9	^57^ ^–^ ^59^
***S. pyogenes***				
Rgg2	DILIIVGG	SHP2	*S. pyogenes* NZ131	^3^
Rgg3	DIIIIVGG	SHP3	*S. pyogenes* NZ131	
RopB	MWLLLLFL	SIP (LCP)	*S. pyogenes* MGAS10870	^11^ ^,^ ^60^
ComR	SAVDWWRL	M1 XIP	*S. pyogenes* M1 SF370, MGAS8232, MGAS10394, MGAS6180, MGAS5055, MGAS9429, ATCC 10782, MGAS2096, MGAS10750, NZ131	^9^ ^,^ ^61^
ComR	EFDWWNLG	M3 XIP	*S. pyogenes* Manfredo, MGAS10270, MGAS315, SSI-1	
***S. pneumoniae***				
Rgg144	EWVIVIPFLTNL	SHP144	*S. pneumoniae* D39	^7^ ^,^ ^62^
Rgg0939	DIIIIVGG	SHP939	*S. pneumoniae* R6, D39	^6^ ^,^ ^7^
Rgg1518	IWSWIQLIWFETWFWG	SHP1518	*S. pneumoniae* D39	^30^
RtgR	AIIFPWGWPI	RtgS1 Type A	*S. pneumoniae* D39, Sp9-BS68	^22^
RtgR	AIIFPWGWSI	RtgS1 Type B	*S. pneumoniae* D39	
***S. mutans***				
PdrA (Rgg1509)	ETIIIIGGG	SHP/SHP1509	*S. mutans* UA159	^28^ ^,^ ^33^
ComR	GLDWWSL	XIP	*S. mutans* UA159	^9^
***S. ferus***				
ComR	GLSWWGL	XIP	*S. ferus* DSM20646	^63^
***S. mitis***				
Rgg0094	DIIIVGG	SHP0094	*S. mitis* CCUG31611	^29^
***S. suis***				
ComR	GNWGTWVEE	Type A XIP	*S. suis* ZY05719	^64^
ComR	GNWGKWTDG	Type B XIP	*S. suis* CZ130302, ZY05719	
ComR	LGDENWWVK	Type C XIP	*S. suis* ZJJX0908005	
**Other streptococcal species**				
RovS	DILIIVGG	SHP1555/SHP152020	*S. agalactiae* NEM316/A909	^20^ ^,^ ^28^
Rgg2	DILIIVGG	SHP2	*S. dysgalactiae* subsp. e *quisimilis* GGS-LT1	^20^ ^,^ ^21^
Rgg	LLLLKLA	SHP	*S. zooepidimicus* ATCC35246	^8^
RopB _sp_	MWLLLLFL	LCP _sp_	*S. porcinus*	^34^
RopB _ss_	MWLILLFL	LCP _ss_	*S. salivarius*	^34^
ComR	VPFFMIYY	XIP _Sve_	*S. vestibularis* F0396	^59^ ^,^ ^66^
ComR	VITGWWGL	ComS	*S. gallolyticus* UCN34	^66^
ComR	LMCTIAR, LMCTIVR	XIP	*S. sobrinus* NIDR 6715-7, NCTC 10919	^67^
ComR	LTAWWGL	_Sin_ComS _9-15_	*S. infantarius* AV2A, 3AG-1, 11FA-1, ATCC BAA-102, CJ18	^68^
ComR	ITGWWGL	_Sma_ComS _9–15_	*S. macedonicus* DSM15879, ACA-DC198, 679	
ComR	LPYFAGCL	sXIP	*S. salivarius* HSISS4	^65^

^**a**^ Transcrip. Reg. stands for transcriptional regulator.

^**b**^ Peptide sequences follow naming conventions as presented in original publications.

^**c**^ Ref. stands for reference.

Other Rgg systems, such as Rgg0182, ^53^ RggC, ^54^ and multiple newly identified Rgg systems that regulate RaS-RiPPs ^18^ are additionally encoded by various strains of *S. thermophilus*. These systems have either been directly demonstrated to rely on SHPs to exert their effects or are theorized to do so. ^52^ ^,^ ^53^ These Rgg regulators target various loci depending on their specific system of regulation. ^52^ Rgg0812 regulates genes involved heat shock adaptation, ^53^ whereas RggC has been reported to impact oxidative stress response, but target genes have not been characterized. ^54^ Finally, several *S. thermophilus* strains, including JIM8232 and CNRZ1066, use Rgg/SHP systems to control the production of downstream RaS-RiPPs. These include RaS-RiPPs such as: streptide (SHP/Rgg _gp_sali_6_), streptosactins (SHP/Rgg _Sthermo_13_), bicyclostreptins (SHP/Rgg _gp_sali_4_), enteropeptins (SHP/Rgg _gp_sali_5_), and ryptides (SHP/Rgg _gp_sali_7_) ( Table 1). ^18^

Interestingly, Rgg/SHPs appear to be overrepresented in *S. thermophilus* compared to other streptococcal species. In a recent study, different strains of *S. thermophilus* were screened for Rggs and similar transcriptional regulators. It was found that *S. thermophilus* strains encode for a high density of Rgg or Rgg-like regulators. ^18^ Of the Rgg/SHP subfamily, half of these were found to be encoded next to ThiF or SAM radical enzymes (presumably RaS-RiPPs). ^18^ ^,^ ^55^ These data indicate that Rggs and thus RaS-RiPPs are highly prevalent in this species. Therefore, the Rgg/SHP subfamily is widespread in *S. thermophilus* with functional systems regulating several biological activities.

### Streptococcus pyogenes

*S. pyogenes* (Group A Streptococcus, GAS) was another one of the first streptococcal species in which Rggs were demonstrated to interact with SHP peptides to act as transcriptional regulators, ^3^ following *S. thermophilus.* ^5^ ^,^ ^57^ In *S. pyogenes,* the first study to examine this compared Rgg transcriptional regulators in *S. pyogenes* and identified two of these Rggs as having a high-level of similarity to each other (55% identical, 75% similar). ^69^ Upon examining the coding region around the Rgg regulators, small, unannotated ORFs that were predicted to encode for 22 and 23 amino acids were identified. Further experimental validation demonstrated that these encoded for **s**hort **h**ydrophobic **p**eptides (SHPs), later renamed as SHP2 and SHP3 ( Table 1) based on their proximity to their specific Rgg regulators. These systems are essential for the induction of target genes and together Rgg/SHPs compose a functional quorum sensing system in *S. pyogenes.* ^3^ Expression of SHPs in *S. pyogenes* NZ131 requires a functional Rgg2, whereas Rgg3 ( Table 1) acts as a transcriptional repressor. ^3^ In this system, SHP pheromones (DI [I/L]IIVGG) require PptAB to export the SHP precursor, a metalloprotease (Eep *)* to enzymatically mature the precursor peptide, and a functional oligopeptide permease (Opp *)* transporter to import the mature SHP. The pro-peptides for SHPs are converted to mature peptides, SHP2-C8 and SHP3-C8, which can then be imported back into the cytoplasm in an Opp-dependent manner to bind to Rgg regulators and drive transcription. ^20^ ^,^ ^62^ ^,^ ^70^ Rgg2 and Rgg3 have been shown to have differential activation of Rgg target genes, including a large biosynthetic operon of unknown function and a locus encoding for a small protein called *stcA.* ^41^ ^,^ ^71^ Rgg2 activates *shp* expression and regulated loci, whereas Rgg3 represses expression of the system by forming an opposing regulatory circuit. ^3^ Rgg2 and Rgg3 have a competitive relationship due to highly conserved overlapping promoter binding sites present in both the *shp2* and *shp3* promoters. When SHPs bind Rgg2 this drives the activation of quorum sensing, but during SHP-limited conditions, Rgg3 predominantly maintains the system in an inactive state. ^23^ ^,^ ^69^ Later structural analyses of Rgg2 and Rgg3 revealed that both proteins could act as transcriptional activators under specific conditions, such as highly increased SHP concentrations. Therefore, Rgg3 is not strictly repressive by mechanism; but acts as a repressor during low-SHP conditions. ^23^

One of the main targets of the Rgg2/Rgg3 system is the gene *stcA.* StcA acts as a cell wall binding protein, confers lysozyme resistance and is necessary for *S. pyogenes* biofilm formation. StcA is secreted, binds to peptidoglycan in the cell wall via electrostatic interactions, and as such localizes to the cell surface. StcA is also thought to potentially function in conjunction with putative S-layer transglutaminases in the cell to form an S-layer, although this has yet to be definitively determined. ^41^ The other target of Rgg2/Rgg3 signaling is a large biosynthetic gene operon, which was recently renamed *qim* ( **q**uorum-regulated **i**mmunomodulatory **m**odification). ^72^ This operon and *stcA* are upregulated during murine skin infection and murine nasal associated lymphoid tissue (NALT) colonization. ^41^ ^,^ ^72^ ^,^ ^73^ A recent report demonstrated that *qim* modifies the cell wall of *S. pyogenes* by adding a unique *N*-acetylglucosamine-linked ribitol that suppresses the innate immune response via an unknown mechanism. The presence of this modification is necessary for full virulence in a murine skin model, as well as preventing NF-κB activation. ^74^

The activation of the Rgg2/Rgg3 quorum sensing pathway and the genes that it regulates helps to provide a survival advantage to *S. pyogenes* during colonization. In a murine skin infection model with QS-active (WT and ∆ *rgg3*) strains, the presence of this system results in significant weight loss, greater bacterial burden, and progressive loss of epithelial barrier integrity with lesions. Compared to QS-active mutants, when mice were infected with QS-null mutant (∆ *rgg3shp2*_GGG_ *shp3*_GGG_) strains started developed crusted lesions, clearing central erythema and had continuous healing from day 5 to 10 post-inoculation. These results demonstrated that an intact Rgg2/Rgg3 system provides advantages for the survival of *S. pyogenes* on skin infection. ^72^ This system also impacts colonization in a murine oropharyngeal model. Constitutive expression of the system results in higher levels of colonization in mice and lower expression of regulatory cytokines. In contrast, deletion of the positive regulator Rgg2 (thus inactivation of the system) cannot establish colonization in mice. ^73^

Other evidence has shown that this system is important for virulence gene expression as well. Deletion of Rgg2 in the M1 serotype results in differential expression of several virulence factors: lower expression of SIC (streptococcal inhibitor of complement), a streptococcal exotoxin H precursor, and higher expression of genes such as *slo, nga*, and *scpA*. Rgg2 deletion also results in attenuation of *S. pyogenes* M1 serotype in an intraperitoneal murine model. ^75^ In *S. pyogenes* NZ131, induction of this system leads to the lower expression of *slo* (streptolysin O) due to increased expression of the *spy49_0460* efflux protein. ^71^ This observation is consistent with the increased expression of genes reported in M1 serotype in the absence of Rgg2. Additionally, proteins such as SpyCEP and M protein have been reported to have decreased expression when the Rgg2/Rgg3 system is active. Thus, it appears that the Rgg2/Rgg3 system is necessary for *S. pyogenes* colonization and correct expression of virulence factors. ^71^

In line with the necessary requirement for Rgg2/Rgg3 for full virulence and colonization, the Rgg2/Rgg3 system suppresses macrophage responses and pro-inflammatory immune responses. Infection of macrophages with a functional Rgg2/Rgg3 system or the system in a QS locked on state suppresses macrophage NF-κB activity, TNF-α, and IL-6 production. This process necessitates live cells, is thought to be an active process, and requires the presence of the *qim* operon. ^40^ ^,^ ^76^ Macrophages infected with QS-locked on strains downregulate inflammatory pathways and upregulate fatty acid beta-oxidation and oxidative phosphorylation pathways in M2 macrophages. Further investigation of this phenotype found that suppression of inflammatory responses via Rgg2/Rgg3 is primarily due to epigenetic regulation and disruption of transcription factor translocation to the nucleus. ^76^

While SHPs are the main way the Rgg2/Rgg3 system is induced, it can also be triggered via metal availability, different carbon sources, and nitric oxide (NO). ^77^ MtsR, a DtxR-family metallorepressor, binds upstream of *shp3* in response to low iron and manganese levels and represses transcription. ^77^ ^–^ ^79^ Mannose availability also impacts the Rgg2/Rgg3 system, but this is modulated in NZ131 by the Mga transcription regulator and a mannose PTS system (PtsABCD). ^80^ In contrast, NO triggers Rgg2/Rgg3 system induction via formation of dinitrosyliron complexes (DNIC) resulting in NO-dependent iron restriction. ^77^ Hence NO’s involvement is linked to the response to low iron conditions. As such, metal and carbon sensing are distinct regulatory systems that converge during SHP pheromone production and Rgg2/Rgg3 activation. ^42^ ^,^ ^77^ ^,^ ^80^

RopB (Regulator of Protease B, *spy49_1691*, also called Rgg) is another Rgg present in *S. pyogenes*. RopB represents a unique class of Rgg regulators that respond to short leaderless peptides (LCPs), termed SIPs in *S. pyogenes.* ^11^ ^,^ ^37^ ^,^ ^81^ ^,^ ^82^ RopB is located adjacent to *speB,* and is required for the activation of *speB,* which encodes the extracellular cysteine protease of streptococcal erythrogenic toxin B. ^83^ RopB directly controls the expression of *speB* by binding to operator elements at the intergenic region between the *ropB* and *speB* transcription start site and drives the transcription of *speB* in a growth-phase dependent manner. ^11^ ^,^ ^83^ ^,^ ^84^ RopB also impacts expression of the autolysin *clpB*, a DNA entry nuclease. ^84^ Due to the targets it regulates, RopB is also important for virulence and colonization. For instance, presence of this system is vital for colonization of the mouse oropharynx, survival in blood, and full virulence. ^11^ ^,^ ^60^ ^,^ ^85^ ^–^ ^88^ Another small peptide besides SIP has been demonstrated to impact RopB activity, called Vfr. Vfr acts as an inhibitory peptide and is thought to interact with RopB, preventing it from binding DNA and thus repressing *speB* transcription. ^89^

The discovery of the Rgg2/Rgg3 system established the use of SHPs as QS signals outside of *S. thermophilus*, while the finding that RopB utilizes a distinct LCP called SIP expanded the field’s understanding of peptide signaling in streptococci. Much of the field’s current understanding of Rgg signaling has been established via the study of these systems in *S. pyogenes* and *S. thermophilus,* which has involved the contribution of multiple groups.

### Streptococcus pneumoniae

Cell-cell communication systems have been extensively studied in *Streptococcus pneumoniae,* which includes TCS and Rgg/SHP systems. The best characterized by far is the TCS ComCDE that induces competence. Competence is one of the major cellular processes induced by peptide based quorum sensing in streptococci, and as such we have included a brief overview and their relationship to Rgg/SHP system later on in the review. Many studies have been written on this subject and we briefly summarize these systems, along with referring the reader to several reviews on the topic.

In terms of Rgg/SHP quorum sensing systems, the study of these in pneumococci dates to only the past decade. Multiple Rgg/SHP systems have been characterized in *S. pneumoniae*. *S. pneumoniae* has been reported to carry anywhere between six to eight Rgg/SHP systems in a single strain, ^30^ ^,^ ^90^ although not all of these have been extensively characterized. We cover the Rggs in which significant functions have been assigned to or for which SHP peptides are known. These have been primarily named based on the locus number assigned to the Rgg of interest and include: Rgg144/SHP144, Rgg939/SHP939, RtgR/RtgS, Rgg1952, and Rgg1518/SHP1518 ( Table 1).

One of the first Rgg/SHP quorum sensing systems characterized in *S. pneumoniae* was the Rgg939/SHP939 system. ^6^ Like other Rgg/SHP systems, it consists of an Rgg transcriptional regulator (Rgg939) and a short hydrophobic peptide (SHP939). The precursor SHP is synthesized within the cell and secreted through a peptide transporter called PptAB, and processed via a membrane protease called Eep, as is seen in most Rgg/SHP systems ( Figure 2). ^32^ ^,^ ^91^ When peptide densities increase, the mature SHP is imported into the cell by the oligopeptide permease (Opp) transporter, which then binds to Rgg939 to activate expression of downstream genes. This Rgg/SHP system continues to function through a positive feedback loop in which Rgg/SHP939 upregulates *shp* expression. ^6^ The Rgg939/SHP939 signaling system induces the expression of 11 genes present in a single transcript downstream of *shp* that comprises variety of essential functions such as *mnaB*, UDP-4-galactose-epimerase, a putative xylose isomerase, an AMP-binding enzyme, lantibiotic and bacitracin transport, lanthionine biosynthesis protein, as well as a lactose transporter. Expression of these genes influences polysaccharide production. Additionally, a *S. pneumoniae* D39 strain that lacks this Rgg has impaired biofilm formation and lower fitness in a murine model of lung infection. ^6^

Rgg1952, also called RggM, was identified prior to Rgg939, but its connection to quorum sensing was not identified at the time of its discovery. ^92^ However, it was demonstrated to be involved in the response to oxidative stress and found to be responsive to oxygen and paraquat levels. Rgg1952 was also found to be necessary for full virulence in a pneumonia model. ^92^ The cognate SHP for this Rgg has not been identified as of yet.

At the same time that the Rgg939/SHP939 system was discovered, another Rgg/SHP was found in *S. pneumoniae*. This was named Rgg144/SHP144. Rgg144 as well as Rgg939 can induce QS in response to sugars found in the respiratory tract, such as galactose and mannose, alongside their native SHP induction. Rgg144 and Rgg939 perform some level of crosstalk, with the presence of Rgg939 and Rgg144 system necessary for full QS induction of each other. Rgg144 and Rgg939 are both important for processes such as mannose metabolism, as well as necessary for pneumococcal colonization in the nasopharynx. ^7^ Rgg144 is also vital for production of a short peptide called the VP1, which has been demonstrated to play a role in pneumococcal colonization and virulence. ^7^ ^,^ ^31^

**Figure 3.**
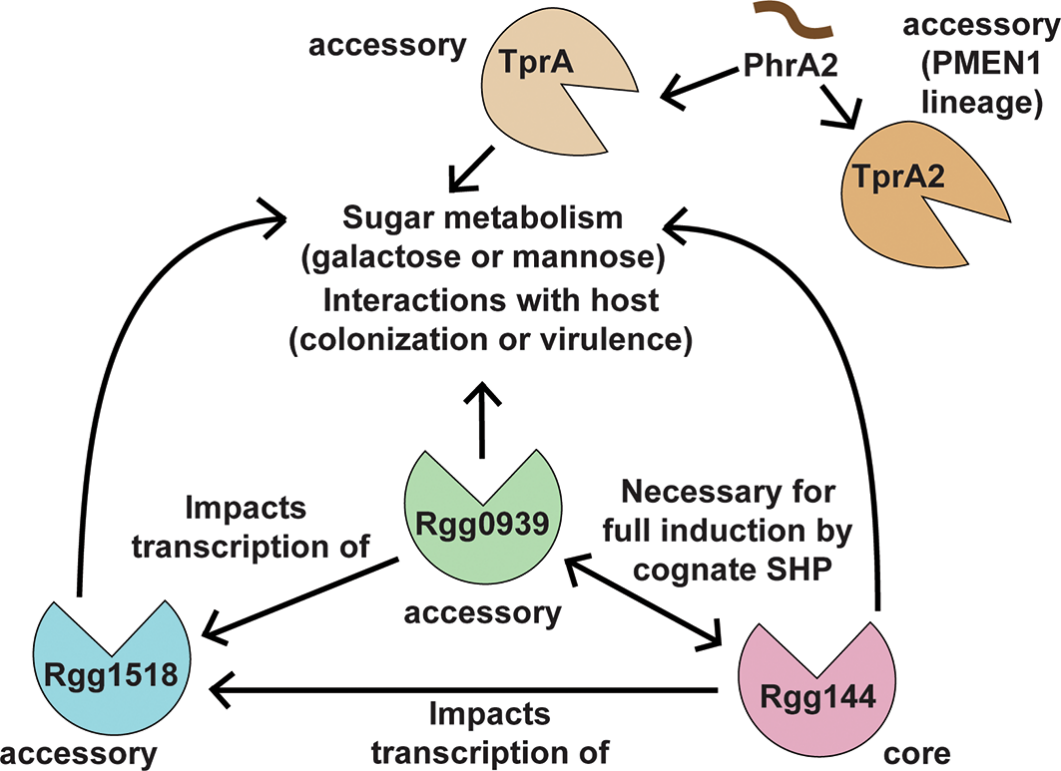
Convergence of Rgg and TprA transcriptional regulators on sugar metabolism and interactions with the host in *S. pneumoniae*. This diagram depicts the current documented interplay between Rgg1518, Rgg144, Rgg0939, TprA, and PhrA2 (of the TprA2/PhrA2 system) and how these regulate sugar metabolism and interactions with the host. Regulators are indicated by if they belong to the core or accessory genome by “core” or “accessory” by each protein. Arrows indicate influence or regulation of a process. Rgg144, Rgg0939, Rgg1518, and TprA all have been documented to influence genes involved in galactose or mannose metabolism, and impact colonization or virulence (interactions with the host), depending on the system. Rgg0939 and Rgg144 transcriptionally regulate the expression of Rgg1518. Rgg144 and Rgg0939 require the presence of each other (in strains where they co-occur) for full induction by their cognate SHP peptides. PhrA2, a peptide produced as part of the TprA2 system, can induce either TprA or TprA2 to elicit downstream functions. TprA regulates galactose and mannose metabolism and is necessary for growth in the respiratory tract.

The Rgg1518/SHP1518 system is another Rgg/SHP system that has been characterized in *S. pneumoniae*. This system has also been implicated in pneumococcal virulence and is responsive to sugars such as galactose and mannose, a common theme in *S. pneumoniae* Rgg/SHP systems. Strains that lack this Rgg have lower growth yields and extended lag phases when grown on galactose and mannose as primary carbon sources. As such, Rgg1518, Rgg144, Rgg939 and another QS regulator called TprA all appear to impact sugar metabolism (mannose and/or galactose) and interactions with the host (influencing virulence or colonization) ^6^ ^,^ ^7^ ^,^ ^30^ ^,^ ^93^ ^,^ ^94^ ( Figure 3). TprA and its cognate peptide PhrA represent distinct QS regulatory systems from Rgg/SHP systems, but regulate also sugar utilization, neuraminidase activity, lantibiotic expression, and virulence. ^93^ ^,^ ^94^ Some pneumococcal strains contain a second discrete TprA/PhrA system (TprA2/PhrA2) that can induce the TprA/PhrA independently, thereby presumably impacting these phenotypes ( Figure 3). ^95^ Interestingly, transcription of Rgg1518 is impacted by the presence of Rgg144 and Rgg939 ( Figure 3). Rgg1518 acts as a negative repressor of capsule synthesis, as deleting this gene results in increased capsule polysaccharide in the presence of galactose. Finally, it has been demonstrated to directly impact pneumococcal colonization, as a deletion of *rgg1518* results in significantly lower CFU/mL in the nasopharynx of a murine pneumococcal colonization model. ^30^ One target of this system, *spd_1513-1517* has been reported to be influenced by the presence of at least four other Rggs in *S. pneumoniae* D39 (Rgg112, Rgg1786, Rgg1916, and Rgg1952), ^90^ demonstrating crosstalk between Rggs in this species. This crosstalk highlights the potential of Rgg-mediated quorum sensing systems to function as interconnected regulatory networks converging on shared phenotypes or genes.

Finally, RtgR/RtgS (hereafter RtgR/S) is an Rgg/SHP-like system found in pneumococcus that impacts nasopharyngeal colonization. This system belongs to the same family of regulatory systems as Rgg/SHP and ComR/S systems found in streptococci and controls the expression of the *rtg* locus ( **R**gg-regulated **t**ransporter of double glycine peptides) which encodes for peptidase-containing ABC transporters (PCAT) and *rtgS*. RtgS is similar to SHPs and XIPs (peptides involved in induction of competence in streptococci) in most aspects except it lacks a conserved aspartate or glutamate residue. The presence of this system in pneumococci has been demonstrated to confer a survival advantage: wild-type strains with RtgR/S outcompeted strains that lacked the system during nasopharyngeal colonization in mice. ^22^

Thus, *Streptococcus pneumoniae* harbors several Rgg/SHP-like transcriptional regulatory systems that play important roles in pneumococcal physiology such as virulence, colonization, biofilm formation, and sugar metabolism. Some of the Rgg/SHP regulators have interlinked functions possessing cognate pheromone inducers present in the core and accessory genome driving specific functions that integrate metabolic state and environmental or fitness cues. Together, these Rgg systems illustrate that pneumococcus has a diverse set of Rgg-like transcriptional quorum sensing systems to adapt to host niches and coordinate community behaviors.

### Streptococcus gordonii

While Rgg/SHP systems as quorum sensing systems were established in *S. pyogenes* and *S. thermophilus,* ^3^ ^,^ ^5^ Rgg proteins themselves had already been defined as transcriptional regulators. In fact, the Rgg family was first named as **r**egulator **g**ene of **g**lucosyltransferase, *gtfG,* in *S. gordonii* as a member of a family of streptococcal positive regulatory genes. ^96^ ^,^ ^97^ It was demonstrated in this species that the glucosyltransferase gene, *gtfG*, catalyzes the formation of glucan from sucrose, and is regulated via a positive transcriptional regulatory determinant that the authors named *rgg,* as well as a putative protein designated as *rggD.* Both Rgg and RggD were found to have a similar helix-turn-helix domain at the N-terminus and 220 amino acids region rich in alpha helices at the C-terminal region, suggesting they belonged to the same family of transcriptional regulators. Despite having structural similarity, Rgg and RggD functionally differ. Rgg is an established positive regulator of *gtfG* expression, whereas RggD does not measurably affect *gtfG* activity, impact the production of *gtfG* polycistronic transcripts, or alter GTF (glucosyltransferase) levels. RggD was also found to have no detectable effect on the transcription of adjacent or downstream genes. This suggests that *rggD* might control a separate operon rather than its adjacent locus, in contrast to Rgg which directly controls *gtfG* expression. ^98^ ^–^ ^100^ Minimal work on these systems in *S. gordonii* outside of their impact on glucosyltransferases has been performed. Orthologs of these were later found in *S. pyogenes* and *S. thermophilus* to rely on SHPs to exert their activities, but *S. gordonii* was the first organism in which Rggs were defined as transcriptional regulators.

### Streptococcus mutans

Quorum sensing systems that have been best described in *S. mutans* are ones involved in competence development and bacteriocin expression, ^9^ ^,^ ^10^ ^,^ ^101^ including the ComCDE and ComR systems, as discussed later on in the review. Rgg/SHP systems, while related to these quorum sensing systems, have not been studied extensively in this organism.

One Rgg/SHP quorum sensing system has been investigated in this organism, that regulates a specialized biosynthetic operon producing a RaS-RiPP. In *S. mutans* UA159, the Rgg/SHP in question encodes a SHP in a small open reading frame adjacent to the Rgg. This was originally discovered by Fleuchot et al. and functions as an Rgg/SHP system when placed into the *S. thermophilus* LMD-9 background. ^28^ In the study examining its connection to RaS-RiPP induction, the gene was re-named PdrA (Rgg1509; Table 1) for pheromone dependent regulator of RiPP. The operon regulated by PdrA was found to encode for the RaS-RiPP named Tryglysin B (TryB). ^33^ Like other Rgg/SHP systems, induction of the RaS-RiPP operon relies on the presence of SHP and similar export and import mechanisms. ^28^ ^,^ ^33^ Tryglysins are the founding members of a new subclass of RiPPs in *S. mutans* and the related species *S. ferus*. These peptides are ribosomally encoded and then modified to create a tetrahydro[5,6]benzindole motif. ^26^ These 7-mer peptides have bacteriostatic activity towards other streptococci with complete growth inhibition of *S. mitis, S. oralis, S. pneumoniae,* and *S. sanguinis* at 100 nM tryglysin treatment. However, the mechanism of tryglysin-mediated inhibition is unknown. ^33^

### Streptococcus ferus

While *S. ferus* is known to encode for several Rgg/SHP systems, including the tryglysin production system, ^26^ ^,^ ^33^ little is known about how these systems function in this organism. Several proteins with similarity to Rgg and ComR regulators have been observed in *S. ferus* genomes. In the type strain of *S. ferus* (DSM 20646), this includes four ComR/Rgg like proteins: a canonical competence regulator *comR* (A3GY_RS0108865), a secondary ComR-like protein *comR2* (A3GY_RS0106270), *rggA* (A3GY_RS0105975), and *pdrA* (A3GY_RS0100490), which regulates the Rgg/SHP system involved in tryglysin biosynthesis. ^63^ ComR is the main competence regulator in *S. ferus*, relies on XIP induction, and behaves similarly to other ComR systems. ^63^ While it is known that *S. ferus* produces tryglysin A (TryA), if this Rgg/SHP system functions similarly to the *S. mutans* ortholog has not been thoroughly characterized. As such, much remains to be discovered concerning Rgg/SHP regulation in this species.

### *Streptococcus agalactiae* and other streptococci

*S. agalactiae* (GBS, or Group B Streptococcus) is another species demonstrated to utilize Rgg/SHP quorum sensing to control aspects such as virulence and interspecies communication. GBS species carry an Rgg2 ortholog called RovS and its associated small peptide SHP1520, which is regulated and induced in a similar manner to other Rgg/SHP systems. ^32^ This system performs inter-species crosstalk, with SHP1520 being able to activate the Rgg2/3 system in GAS, and the RovS/SHP1520 system being able to respond to similar SHPs from GAS and *S. porcinus.* ^20^ This crosstalk has been shown to occur during co-culture of GAS and GBS, and SHP1520 results in increased biofilm of GAS under in-vitro conditions. ^20^ In addition to serving as a system mediating interspecies communication, RovS/SHP1520 forms a functional QS circuit in GBS, is highly conserved, and plays an important role in GBS virulence. ^20^ ^,^ ^32^ ^,^ ^39^ In particular, the system is important for persistence in the liver and spleen, with disruption of *shp* and *rovS* genes resulting in decreases in adherence and invasion of human HepG2 hepatic cells. ^32^ Targets of this system include the gene *gbs1556,* a putative transglutaminase/protease enzyme involved in growth in human amniotic fluid and GBS infection of macrophages, and *fbsA,* an adhesin involved in binding to fibrinogen. ^32^ ^,^ ^102^ ^–^ ^104^ As such, the RovS/SHP system contributes to GBS virulence and bacterial pathogenesis via regulation of several targets.

Multiple other streptococcal species utilize the Rgg/SHP-type quorum sensing systems, suggesting a widespread role of this system in communication. This review cannot cover all defined systems present in streptococci, but we mention several additional species of relevance here. This includes *Streptococcus dysgalactiae* subsp. *equisimilis,* ^20^ S *. macedonicus,* ^20^ *S. infantarius,* ^20^ *S. porcinus,* ^20^ and *S. zooepidemicus* ^8^ ( Table 1).

### A brief overview of natural competence in streptococci

We now briefly discuss natural competence, given these systems’ relevance to quorum sensing in streptococci and extensive history of study. ComCDE and ComR are peptide-dependent quorum sensing regulators that induce competence in various streptococcal species; however, they operate quite differently from each other and are considered distinct from Rgg/SHP systems. ComR regulators are part of the RRNPP regulator superfamily, are structurally similar to Rgg/SHP systems, and are typically included in analysis examining the incidence of Rgg/SHP systems in streptococci given their relevance to peptide signaling ^16^ ^,^ ^18^ ^,^ ^28^ However, they use distinct signaling peptides ( Table 1), and induce genes involved in competence. ComCDE and ComRS systems both hinge on the regulation of *sigX* expression (also known as *comX*), an alternative sigma factor critical for genetic transformation in streptococci. ^105^ ^,^ ^106^ Streptococci have been demonstrated to possess either ComCDE, ComR, or both of these systems, depending on the species. We discuss this briefly in the two best-characterized species for each of these systems: *S. pneumoniae* for ComCDE and *S. mutans* for ComR.

**Figure 4.**
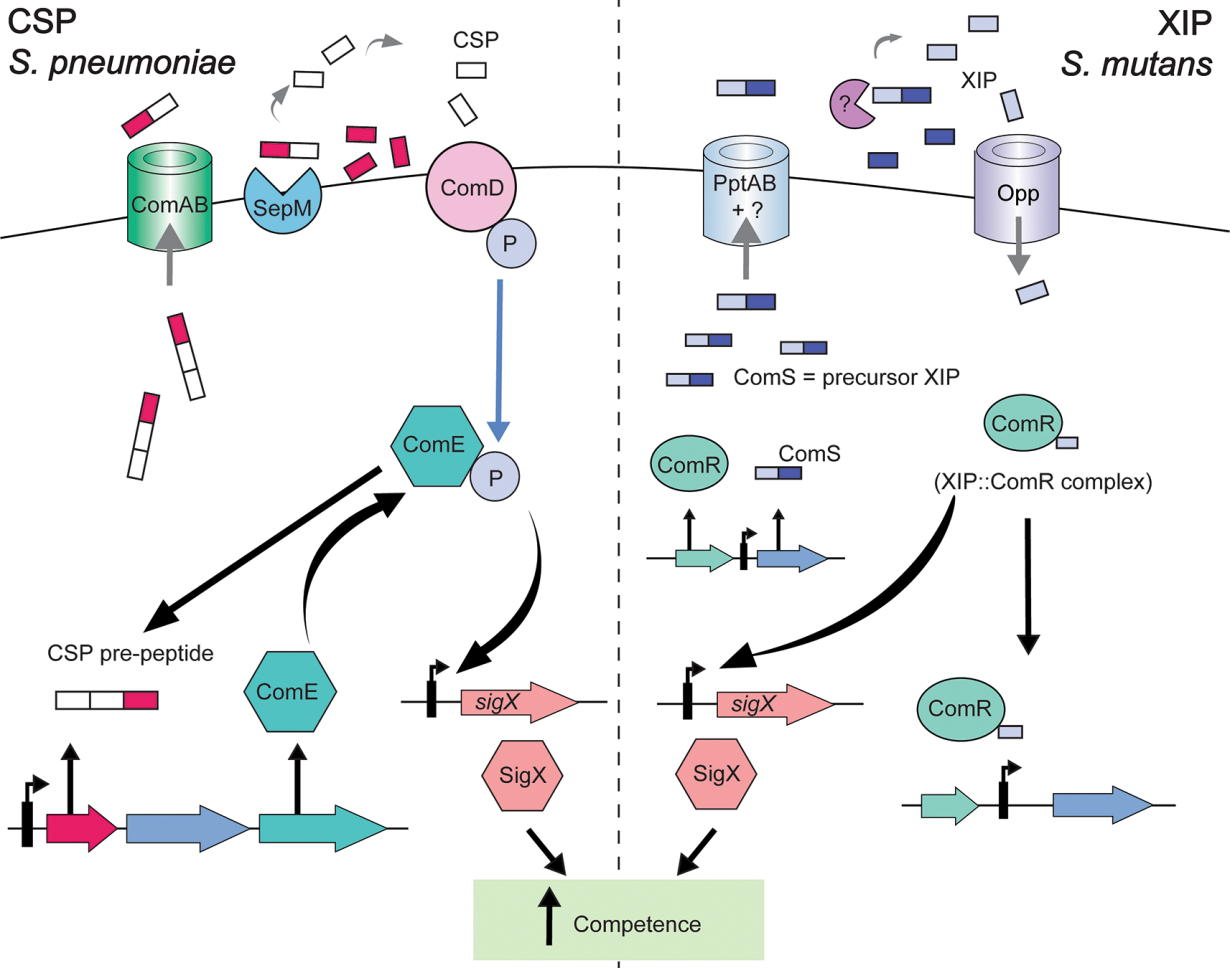
An overview of the competence mechanism utilizing CSP and XIP in streptococci, with *S. pneumoniae* and *S. mutans* as prototypical examples. Both systems utilize a small peptide signal that is secreted extracellularly to induce the process of competence and rely on the alternative sigma factor SigX (also known as ComX) to induce genes necessary for competence. In *S. pneumoniae*, the ComC precursor CSP peptide is exported outside of the cell through ComAB. Peptides are trimmed by SepM and CSP is sensed by ComD in the cellular membrane. ComD is phosphorylated, and phosphotransfer to ComE occurs. Phosphorylated ComE upregulates *sigX* and which leads to the induction of competence related genes. In *S. mutans*, ComS (XIP precursor) is produced from the ComRS locus and exported through PptAB. ComS peptides are trimmed to form XIP, although the exact mechanism is unknown (indicated by ?), and imported back into the cell through Opp. Intracellular signaling by ComS has also been demonstrated to occur. XIP binds to ComR, resulting in activation and upregulation of competence gene expression, due to induction of the alternative sigma factor SigX. Arrows in the figure indicate the following: larger black arrows indicate induction of a protein/peptide or regulation by a protein or protein complex. Small black arrows located on gene diagrams indicate promoters. The light blue arrow indicates transfer of a phosphate (specifically from ComD to ComE). Light gray arrows indicate processing of a peptide or movement of a peptide through a protein complex.

In streptococci, the activity of SigX can be modulated by one of by two signaling pathways, ComCDE or ComRS, that respond to **c**ompetence **s**timulating **p**eptide (CSP) and **S**igX- **i**nducing **p**eptide (XIP), respectively ^9^ ^,^ ^105^ ^,^ ^107^ ( Figure 4). For the ComCDE system, competence is initiated via the binding of CSP to ComDE. ^108^ ^,^ ^109^ When CSP is secreted and at high density outside the cell, it binds to ComD. Once sensed, ComD undergoes autophosphorylation and the phosphorylation signal is transmitted to the cognate response regulator ComE ( Figure 4). ^109^ ^,^ ^110^ This activation induces the expression of *sigX* and as a result expression of competence related genes. ^105^ ComCDE systems have been the subject of intense study for approximately a century, dating back to the initial studies by Frederick Griffith in 1928 demonstrating that a transforming principle existed in *S. pneumoniae* that could lead to the metamorphosis of avirulent rough strains to virulent smooth strains in mice. ^111^ *S. pneumoniae* was the first organism in which CSP was characterized, as well as the necessity for processing of ComC precursor peptide to produce functional CSP, and the use of the ComCDE TCS in this process. ^12^ ^,^ ^112^ It was also the first organism in which SigX was found to be necessary for expression of competence genes. ^105^ ^,^ ^113^ ^,^ ^114^ As such, this species has served as the foundation for studies of competence involving ComCDE systems.

The second way that competence induction can occur in streptococci is via the ComRS pathway, with *S. mutans* as the prototypical example ( Figure 4). This is comprised of ComR and the XIP signaling peptide (encoded by *comS*). The XIP (Sig **X**- **i**nducing **p**eptide) is 7 amino-acids long and is derived from the C-terminal region of a 17 amino-acid precursor ComS peptide. The precursor ComS peptide is translated, exported to the extracellular space, and cleaved by proteases to produce XIP. XIP is then re-imported into the cell via the oligopeptide transporter Opp. When inside the cell, XIP binds to ComR regulator to drive the transcriptional expression of *sigX* and *comS* promoters. XIP binding to ComR additionally induces a positive feedback loop that amplifies the transcription of ComS, thereby producing increased levels of ComS/XIP ( Figure 4). ^9^ ^,^ ^58^ ^,^ ^66^ ^,^ ^115^ ^–^ ^117^ ComR binding to XIP in turn induces the expression of *sigX* and competence related genes. ^9^ Intracellular signaling via ComRS has also been documented. ^10^ Interestingly, while *S. mutans* does possess a ComCDE system, it does not directly induce competence. Induction of competence in *S. mutans* via addition of CSP is indirect, thought to be via cross-talk with the ComRS system, and is media dependent. ^118^ ^,^ ^119^ Instead, the ComCDE system is primarily responsible for controlling bacteriocin expression in this species. ^120^

As previously mentioned, how these systems are integrated is different depending on the streptococcal species. Some species possess both ComCDE and ComRS type systems, while other species contain only a ComCDE or ComRS system ( Table 1). ^9^ ^,^ ^115^ ^,^ ^117^ For example, *S. ferus* and *S. thermophilus* undergo natural transformation but both species only possess the ComRS system ( Table 1). ^57^ ^,^ ^58^ ^,^ ^63^ In contrast, *S. pneumoniae*, the best characterized organism in terms of competence, only contains the ComCDE system. ^121^ There are variations on the ComRS and ComCDE systems from their canonical classification, with different XIP or CSP motifs ( Table 1, for brevity only XIP sequences are listed) and different expression profiles seen in species such as *S. thermophilus, S. suis, S. mitis, S. anginosus,* and *S. salivarius.* ^5^ ^,^ ^57^ ^,^ ^66^ ^,^ ^68^ ^,^ ^122^ For reviews covering competence in various streptococcal species, we refer the readers to. ^108^ ^,^ ^123^

### RaS-RiPPs as natural products and targets of Rgg/SHP QS

**Figure 5.**
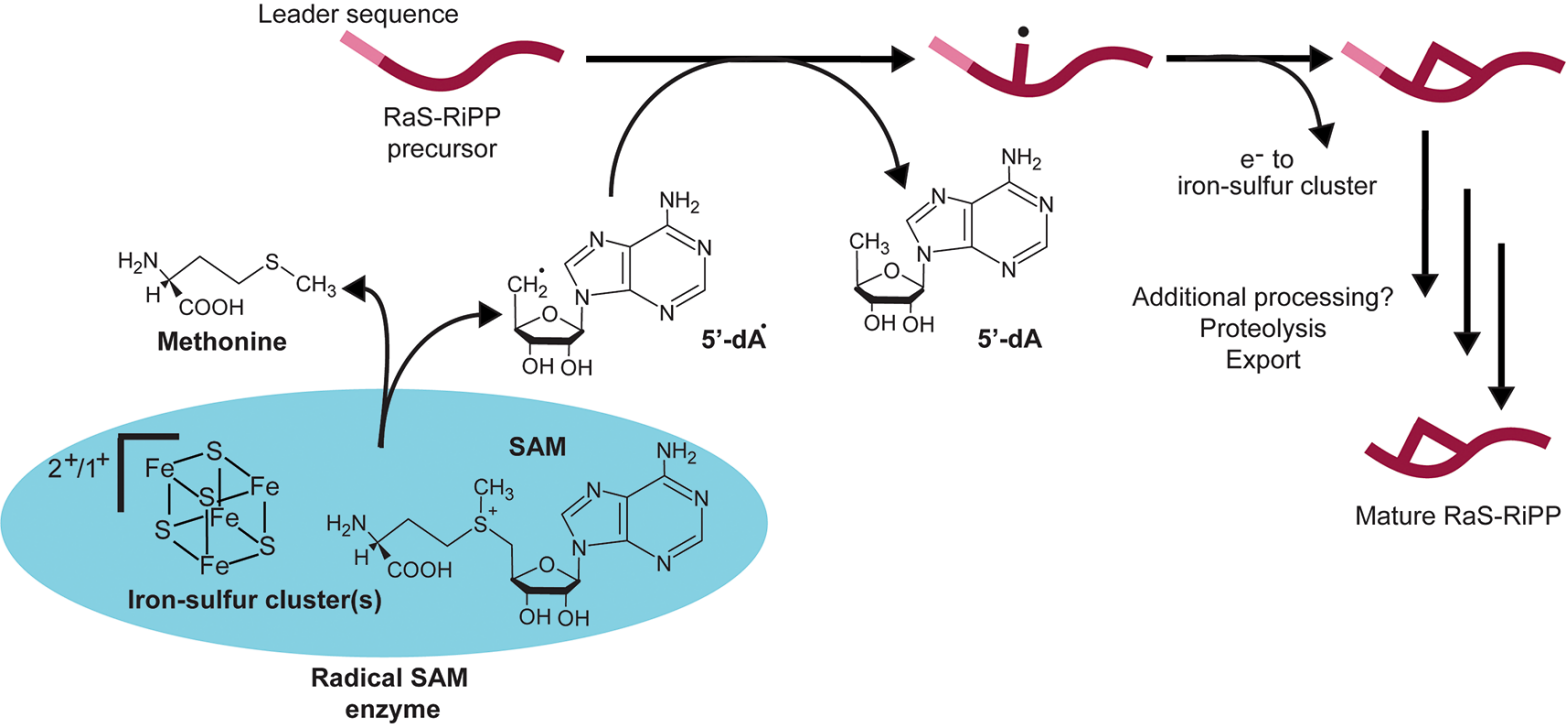
General mechanism of RaS-RiPP biosynthesis. In this general mechanism, the RaS-RiPP precursor peptide is synthesized by the ribosome. This precursor contains a leader sequence that will be removed later during proteolytic processing. The precursor is shuttled to the radical SAM (S-adenosylmethionine) enzyme, which contains one or more iron-sulfur clusters [4Fe-4S]. The iron-sulfur cluster interacts with the SAM cofactor to reductively cleave it, generating methionine and a 5′-deoxyadenosyl radical (5′-dA•). This results in oxidation of the iron-sulfur cluster. 5′-dA• interacts with the RaS-RiPP precursor to generate a radical intermediate on the RaS-RiPP, which after subsequent rearrangements, forms a modification on the precursor. This results in the formation of 5′-dA and release of an electron that is donated back to the iron-sulfur cluster in the radical SAM enzyme, restoring it to the reduced state. The modified RaS-RiPP precursor may then undergo additional processing steps, although this varies depending on RaS-RiPP family. The leader sequence from the precursor is cleaved and the mature RaS-RiPP is exported into the extracellular space.

RaS-RiPPs have recently emerged as a large family of natural products regulated by Rgg/SHP systems. RaS-RiPPs are ribosomally translated peptides that are post-translationally modified by RaS enzymes that install complex modifications. ^26^ First classified as a superfamily in 2001, **ra**dical ***S***-adenosylmethionine (RaS) enzymes have been of interest due to their ability to catalyze complex cellular reactions across all domains of life. ^124^ They are considered one of the most versatile biochemical enzyme superfamilies with over 100,000 homologous enzymes. ^125^ The enzymatic function is initiated by a radical reaction in which cofactor SAM binds via its α-amino and carboxylate groups to an iron-sulfur cluster in the RaS enzyme ( Figure 5). This bond is reductively cleaved, typically leading to the production of 5′-deoxyadenosyl radical (5′-dA•), which then forms a radical on the substrate the RaS enzyme interacts with ( Figure 5). RaS enyzmes thus function as cellular methylating agents and donate methyl groups to various acceptors such as DNA, proteins, and other small molecules ( Figure 5). ^125^ The action of SAM methylation can lead to processes within the cell including gene regulation and the biosynthesis of metabolites. ^126^ ^–^ ^128^

In streptococci, RaS enzymes catalyze modifications on their respective precursor peptides during RiPP biosynthesis ( Figure 5). During RaS-RiPP biosynthesis, a precursor peptide composed of a N-terminal region (leader peptide) and a C-terminal region (core peptide) is synthesized by the ribosome, modified by the RaS enzyme and subsequent tailoring enzymes, and undergoes proteolysis and export to form the final natural product ^26^ ^,^ ^43^ ( Figure 5). A seminal study revealed Rggs are linked to RaS-RiPPs and found that streptococci possess 16 distant Rgg/RaS-RiPP subfamilies. These subfamilies are named based on the conserved motifs within their precursor peptide sequences. ^26^ ^,^ ^43^ ^,^ ^44^ RaS-RiPP subfamilies identified include the following: TQQ, WGK, KxxxW, GGG, KGR, HGH, CGx, SSH, KIS, RRR, GRC, QMP, NxxC, NEF, VSA, and CGG. ^26^ ^,^ ^129^ These are defined based on the conserved amino acid sequence present in the core sequence of the RaS-RiPP. We discuss what is known concerning their regulation, individual families, the modifications installed by RaS-RiPPs, and their functions below.

### Chemistry defines RaS-RiPPs

**Figure 6.**
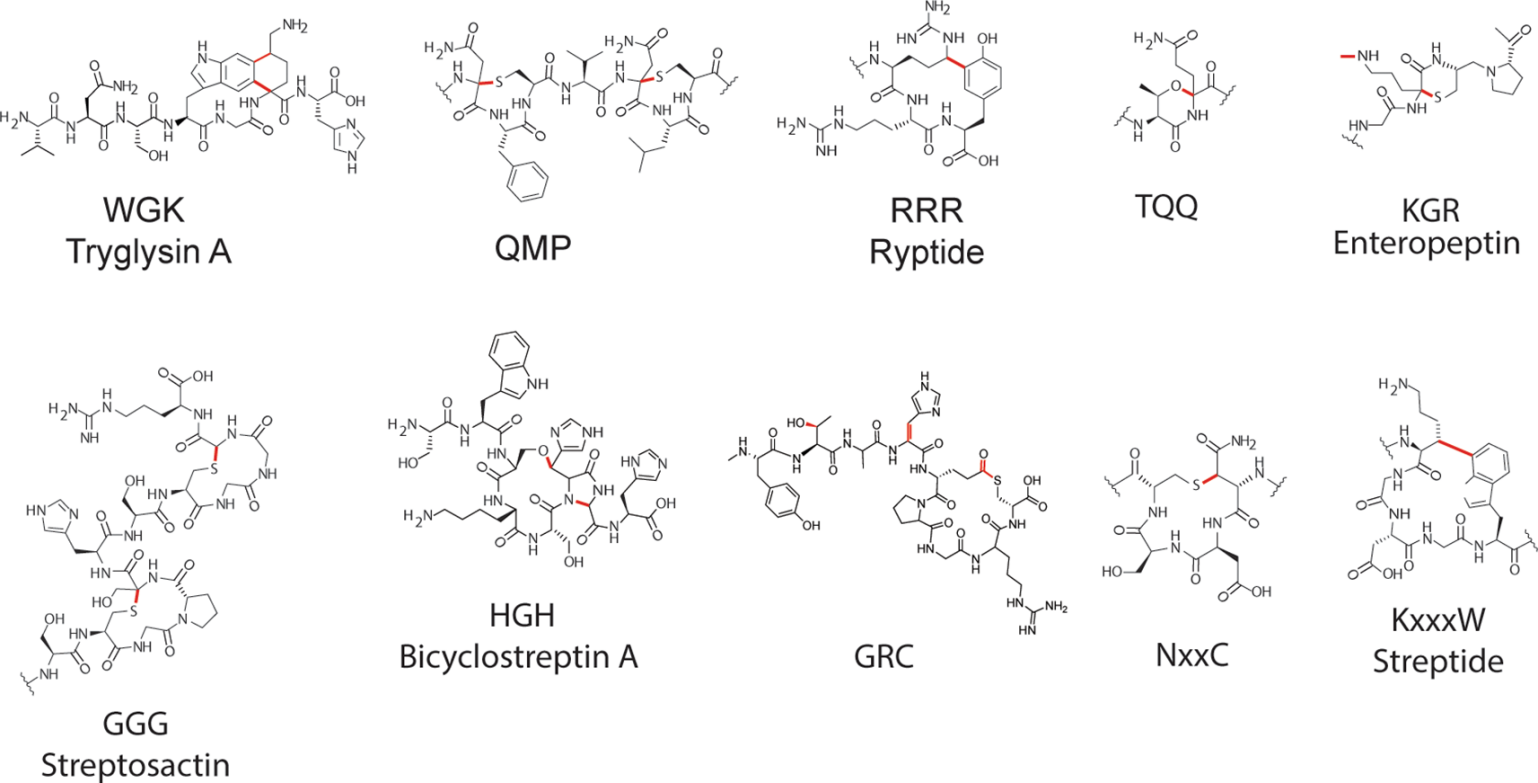
RaS-RiPP structures currently identified or predicted from streptococcal RaS-RiPP subfamilies with the exception of Enteropeptin from *Enterococcus cecorum.* Modifications installed by RaS and additional modification enzymes encoded by respective RaS-RiPP systems are indicated by red chemical bonds in each structure. Squiggle lines indicate where structures are abbreviated for clarity. Subfamilies include WGK (Tryglysin A as an example), QMP, RRR (Ryptides), TQQ (Threoglucins/Rotapeptides), KGR (Enteropeptin, core structure shown), GGG (Streptosactin), HGH (Bicyclostreptin A as an example), GRC, NxxC, and KxxxW (Streptide).

RaS enzymes catalyze various modifications creating unique motifs across numerous superfamilies of RaS-RiPPs ^43^ ^,^ ^129^ ( Figure 6). Modifications that have been found to present in RaS-RiPPs include heterocycles formed by linkages between Lys-Trp residues, β-thioether linkages, and sactionine bridges. We briefly discuss the documented modifications in each family below.

In the WGK and streptide families, biosynthetic gene clusters that encode for tryglysin and streptide (also called KxxxW) possess RaS enzymes that introduce Lys-Trp linkages ( Figure 6). ^26^ ^,^ ^51^ Streptide was the first demonstration of an Rgg-linked RaS-RiPP, although at the time it was not realized how broad this distribution across streptococci truly was. ^50^ ^,^ ^51^ These modifications were the first to be documented as being RaS-RiPPs.

In the QMP and GGG subfamilies, RaS enzymes form sactionine bridges, with resulting RaS-RiPPs having two sactionine bridges present in their structures ( Figure 6). ^47^ ^,^ ^133^ The QMP subfamily yields suisactin, whereas streptococci that contain the GGG subfamily produce streptosactin. Streptosactin was noted to install two consecutive α-thioether crosslinks in a topology that had not been documented previously for sactipeptides, and these crosslinks are installed processively by the RaS enzyme GggB. In contrast, RaS enzymes from the QMP subfamily form unusually small macrocycles that contain only a single intervening residue. As such, both of these yield unique sactipeptides that are distinct from previously elucidated structures. ^47^ ^,^ ^133^

In the TQQ family, the RaS enzyme TqqB forms an ether modification. TqqB catalyzes the formation of the ether cross-link through joining the threonine side chain oxygen to the α-carbon of the adjacent glutamine residue in TqqA, forming threoglucin ( Figure 6). ^130^ Multiple versions of threoglucins have been documented, which all possess the same core modification but have additional amino acids on either the N or C-terminus of the peptide. ^49^ Four variants that additionally contain a hydroxyl group on a C-terminal tyrosine have also been documented. ^49^

In the KGR subfamily, there are currently no known structures from *Streptococcus*; however, structures have been defined from *Enterococcus* termed enteropeptins, which are small sactipeptides containing a thiomorpholine ring ( Figure 6). ^48^ Similar to threoglucins, several versions of enteropeptins have been documented, all containing the same core modification. Enteropeptins differ in the distinct leader or follower sequences they possess outside of the core modification. ^18^ ^,^ ^48^ Documented enteropeptins include enteropeptin A, B, C, and D, arising from *E. cecorum* or *S. thermophilus*.

Within the NxxC subfamily, RaS enzyme NxxcB installs an intramolecular β-thioether bond onto its substrate peptide though the connection of Cys-thiol to the β-carbon of an upstream Asn residue ( Figure 6). This was the first documentation of this type of reaction being carried out by a RaS enzyme. Interestingly, NxxcB was also observed to be able to accept alternative substrates outside of the documented RaS-RiPP precursor, implying it could be harnessed to install non-natural β-thioether bonds. ^134^

In the HGH subfamily, numerous forms of peptides are produced known as bicyclostreptins in which a macrocyclic beta-ether and heterocyclic linkages between backbone amide nitrogen and adjacent alpha-carbon are formed ( Figure 6). Similar to other RaS-RiPP families, these peptides contain the same core modification and are distinct based on the number of additional amino acids at the N-terminus of the peptides. *S. agalactiae* and *S. thermophilus* are both documented producers of bicyclostreptins. ^46^

Several other modifications have been observed in RaS-RiPP families, such as the RRR family in which peptides are modified with an arginine-tyrosine crosslink, ^132^ or the GRC family in which peptides are modified to contain a C-terminal Glu-to-Cys thiolactone macrocycle, L- *allo*-Thr, and didehydrohistidine. ^129^ All of the characterized structures of mature RaS-RiPP products have been elucidated through the work of the Seyedsayamdost laboratory ( Figure 6).

### RaS-RiPPs with antimicrobial properties

Some subfamilies of Ras-RiPPs have been found to possess inhibitory properties ( Table 2). Members of the WGK RaS-RiPP subfamily, Tryglysin A and Tryglysin B produced by *S. ferus* and predicted in *S. mutans,* have bacteriostatic activity towards other streptococci. ^33^ *S. mutans* is a streptococcal species that results in cavities in humans, while *S. ferus* was isolated from the oral cavity of rats and later isolated from pigs. ^135^ ^,^ ^136^ Tryglysins (TryA and TryB) inhibit the growth of other streptococci such as *S. oralis*, *S. sanguinis*, *S. pneumoniae* at 100 nM concentrations. ^33^ Due to *S. mutans* and *S. ferus’* involvement in the oral cavity, it stands to reason that tryglysin could be used by these species to interact with oral communities. A recent study took the first steps in defining the impact of TryA on *ex-vivo* oral microbiomes. It was found that a saliva derived oral inoculum had delayed growth and acidification in a chemically defined media (CDM) upon addition of tryglysin compared to control conditions. Shotgun metagenomics revealed that growth in CDM resulted in the streptococcal species *S. salivarius* dominating the culture under anaerobic conditions. Tryglysin addition was marked by a concomitant increase of *Saccharibacteria.* ^45^ However, due to the inactivity of tryglysin under typical saliva culturing conditions, findings were limited. Further testing within a medium that can support a wide variety of oral species and tryglysin activity will be needed to understand interaction with oral species.

**Table 2.** Experimentally established or predicted RaS-RiPPs and their functions.

Peptide	Producer Streptococci	Function	Reference
**Threoglucins/Rotapeptides (TQQ)**			
Threoglucins	*S. suis*	Growth inhibition of *S. suis*	^49^
Other threoglucins	*S. suis, S. suis sv., S.* *azizii*	Function not characterized	^26^ ^,^ ^130^
**Tryglysins (WGK)**			
Tryglysin A	*S. ferus*	Growth inhibition of *S. ferus and* other streptococcal species	^33^
Tryglysin B	*S. mutans*	Growth inhibition of *S. mutans* and other streptococcal species	^33^
Other tryglysins	*S. equi zooepidemicus, S. equinus, S. ferus, S. mutans, S. sp.*	Function not characterized	^26^
**Streptides (KxxxW)**			
Streptide (also called Pep1357C)	*S. thermophilus*	Function not characterized	^50^ ^,^ ^51^
Other streptides	*S. agalactiae, S. mitis, S. suis, S. thermophilus*	Function not characterized	^26^ ^,^ ^131^
**Streptosactins (GGG)**			
Streptosactin	*S. thermophilus*	Putative fratricidal agent	^47^
Other streptosactins	*S. constellatus pharyngis, S. gordonii, S. oralis oralis, S. oralis tigurinus, S. parasanguinis, S.* spp. *, S. thermophilus*	Function not characterized	^18^ ^,^ ^26^
**Enteropeptins (KGR)**			
Enteropeptin A	*Enterococcus cecorum*	Growth inhibition of *E. cecorum* producer strain	^48^
Enteropeptin B	*Enterococcus cecorum*	Function not characterized	^48^
Enteropeptin C	*Enterococcus cecorum*	Function not characterized	^48^
Enteropeptin D	*S. thermophilus*	Function not characterized	^18^
**Bicyclostreptins (HGH)**			
Bicyclostreptin A	*S. thermophilus*	Growth inhibition of *S. thermophilus* producer and other strains	^46^
Bicyclostreptin B	*S. thermophilus*	Function not characterized	^46^
Bicyclostreptin C	*S. agalactiae*	Growth inhibition of *S. thermophilus*	^46^
Other bicyclostreptins	*S. agalactiae, S. equi zooepidemicus, S. intermedius, S. mitis, S. thermophilus*	Function not characterized	^18^ ^,^ ^26^
**CGx**			
CGx	*S. equi ruminatorum, S. mitis, S.* spp. *, S. suis sv., S. thermophilus*	Function not characterized	^26^
**SSH**			
SSH	*S. equi zooepidemicus, S. mitis, S. parasanguinis, S.* spp.	Function not characterized	^26^
**KIS**			
KIS	*S. suis*	Function not characterized	^26^
**Ryptides (RRR)**			
Ryptides	*S. parauberis, S. suis*	Function not characterized	^26^ ^,^ ^132^
**GRC**			
GRC	*S. pneumoniae, S. oralis*	Function not characterized	^26^ ^,^ ^129^
**QMP**			
Suisactin	*S. suis*	Function not characterized	^26^ ^,^ ^133^
**NxxC**			
NxxC	*S. orisratti*, *S. porci, S. equi zooepidemicus*	Function not characterized	^26^ ^,^ ^134^
**NEF**			
NEF	*S. mitis, S. marmotae*	Function not characterized	^26^
**VSA**			
VSA	*S.treptococcus* spp.	Function not characterized	^26^
**CGG**			
CGG	*S. orisratti*	Function not characterized	^26^

Other research in streptococci has been focused on the sactipeptide termed streptosactin (GGG subfamily). Streptosactin, consisting of a 14-mer peptide with a pair of 4-residue sactionine macrocycles, inhibits growth of the producing host, *S. thermophilus*, with 1 μM of streptosactin causing complete growth inhibition. Streptosactin biosynthesis is correlated with the expression of early competence genes, and as such it has been proposed that streptosactin is the first fratricidal agent in *S. thermophilus.* This is primarily due to its ability to effectively exhibit self-killing activity as well as an observable cell-clumping when streptosactin is present. ^47^ ^,^ ^137^ ^,^ ^138^

Threoglucins (TQQ subfamily, also called rotapeptides) are inhibitory towards their producer species *S. suis* at 500 nM. They do not appear to impact the growth of other streptococcal species, ^49^ but modulate the sensitivity of *S. suis* to other antibiotics. For instance, simultaneous application of 2 μM threoglucins A/B with 200 μM ciprofloxacin resulted in significantly higher viability than 200 μM ciprofloxacin alone. This reveals the potential for threoglucins to serve as a growth-curbing signal while allowing *S. suis* to increase tolerance towards toxins or antibiotics. ^49^

Bicyclostreptins (HGH subfamily) are another class of RaS-RiPPs for which bacteriostatic activity has been observed. These have been isolated from culture supernatants of probiotic *S. thermophilus* as well as *S. agalactiae* at nanomolar concentrations. Several variants of bicyclostreptins have been documented, with Bicyclostreptin A and B isolated from *S. thermophilus*, and Bicyclostreptin C was isolated from *S. agalactiae.* ^46^ Bicyclostreptins have bacteriostatic activity against some *S. thermophilus* strains, as well as their producing hosts. Activity of Bicyclostreptin C can be overcome by producer species, as application does not result in a permanent growth inhibition, suggesting that this peptide is degraded, resistant strains can emerge, or subpopulations of immune producer cells can arise. ^46^

Finally, enteropeptins (KGR subfamily), also have narrow-spectrum bacteriostatic activity. Enteropeptin A specifically inhibits the growth of *E. cecorum*, but not other bacterial species such as *S. thermophilus or E. faecalis*. At physiological production levels (1 μM) *E. cecorum* could recover from enteropeptin inhibition, but higher concentrations were inhibitory at least out to 18 hours of growth. Again, the mechanism of inhibition is unknown, and the exact reasons for production unclear. ^48^ *S. thermophilus* also produces enteropeptins, specifically enteropeptin D, but its function is unknown. ^18^

Further research on growth inhibition mechanisms and interactions with bacterial species of the aforementioned RaS-RiPPs and other unexplored families is needed to establish their role in cell physiology and mechanisms of action.

### RaS-RiPPs’ and Rgg/SHP interplay

**Figure 7.**
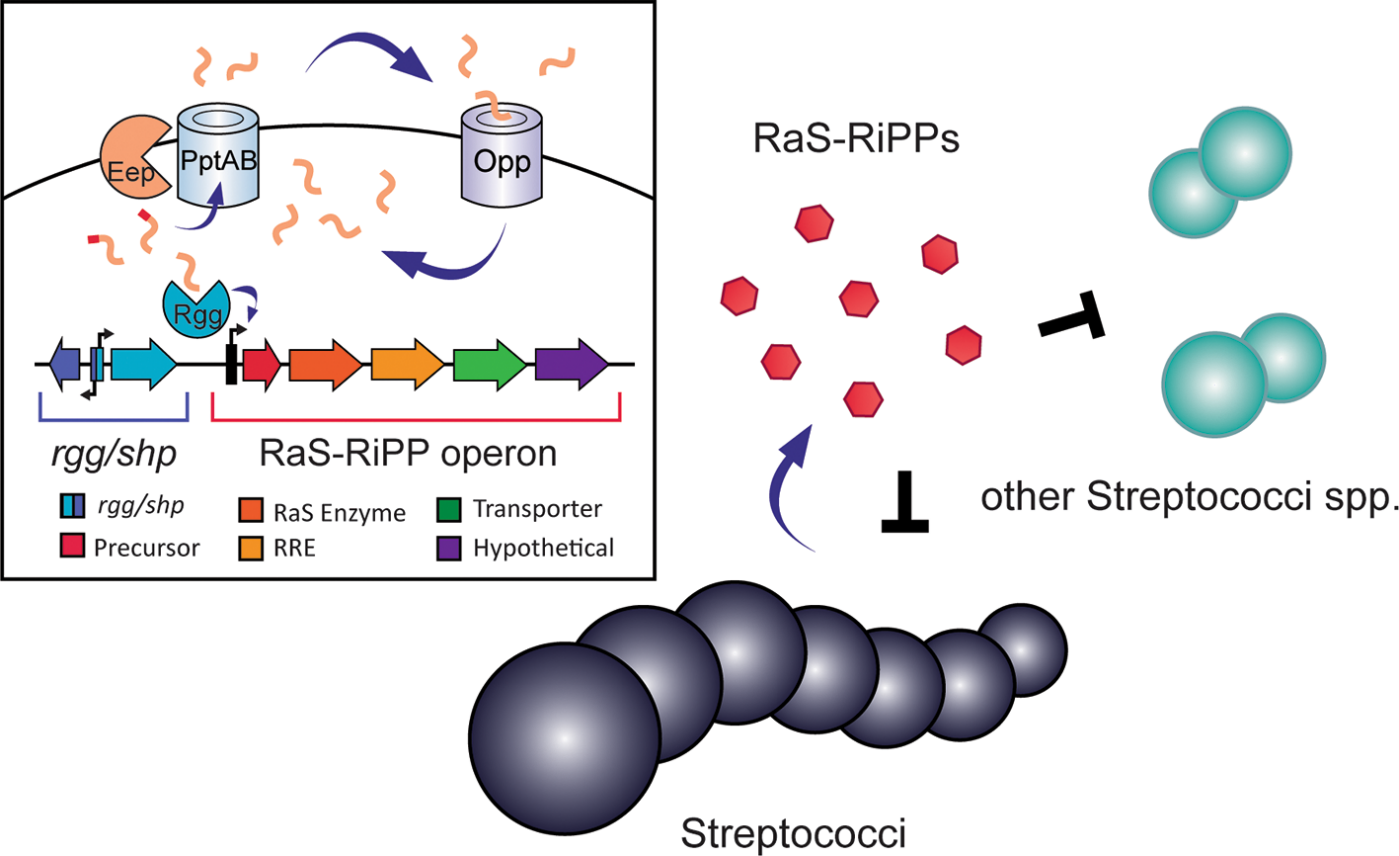
Rgg/SHP quorum sensing in streptococci and RaS-RiPP induction. SHP precursor peptides are trimmed by Eep or alternative peptidases and transported into the extracellular matrix through PptAB. Modification to the peptide is known to occur in some cases in the extracellular matrix. The mature peptide is transported back into the cytoplasm through the Opp transporter where it can bind to Rgg. The Rgg/SHP complex binds to the RaS-RiPP operon and drives the expression of RaS-RiPP natural products. A RaS-RiPP operon can consist of genes including (from left to right) a peptide precursor, RaS enzyme, RiPP Recognition Element (RRE), transporter, and occasionally hypothetical genes. Components of operons can vary between subfamilies, with some lacking certain genes, having additional modification enzymes or possessing two RaS enzymes. Some RaS-RiPP natural products have been demonstrated to inhibit growth of other streptococci, the producing species, or serve as growth regulatory signals.

As previously described, Rgg/SHP quorum sensing systems play an important role in the production of RaS-RiPPs. The first demonstrated example of Rgg/SHP regulation of RaS-RiPPs was from the streptide family: in *S. thermophilus*, streptide production is driven by an Rgg/SHP system that is triggered by high cell density. ^50^ Another system that has been shown to be Rgg/SHP QS dependent is the tryglysin operon in the species *S. mutans.* ^33^ Further supporting this link between Rgg transcriptional regulators and RaS-RiPPs, a recent study demonstrated that several RaS-RiPP operons are modulated by their cognate Rgg/SHP systems in *S. thermophilus.* ^18^ While most RaS-RiPP families are predicted to be controlled by a Rgg/SHP system, there are exceptions, with CGx, CGG, and VSA not possessing an identified *shp* despite having an associated *rgg.* ^26^ However, production of RaS-RiPPs typically correlates with cell density, providing further evidence for Rgg/SHP regulation of these systems. As such, the current model of RaS-RiPP induction is that when a high cell density is present, SHPs are produced and imported into cells, bind to their cognate Rgg transcriptional regulator, and induce expression of a RaS-RiPP operon ( Figure 7). For example, streptosactin and bicyclostreptin production in *S. thermophilus* are cell density dependent, as is Tryglysin A from *S. ferus*, and TQQ from *S. suis.* ^18^ ^,^ ^33^ ^,^ ^46^ ^,^ ^47^ ^,^ ^130^

As such, the use of Rgg/SHP systems control RaS-RiPP systems in streptococci appear to be a conserved mechanism used throughout the genus. It stands to reason that Rgg transcriptional regulators have evolved to be pervasive throughout streptococci and have been co-opted to regulate many of their processes that are necessary for environmental survival, RaS-RiPP production being one of them.

## Conclusion

Streptococci produce an array of small peptides that underly complex reactions in the cell. Although some streptococcal systems are vastly understudied, quorum sensing systems in general have been researched due to their significance to cellular processes. We discuss Rgg/SHP quorum sensing systems which are conserved throughout streptococcal species. These systems can control cellular colonization, virulence, biofilm formation, and even important metabolic programs. ^1^ ^,^ ^3^ ^–^ ^8^ ^,^ ^11^ Importantly, Rgg/SHP quorum sensing systems also regulate the production of streptococcal RaS-RiPPs. Streptococcal RaS-RiPPs are novel products that appear to have multifactorial effects, including inhibiting the growth of other streptococcal species, narrow spectrum activity towards strains of the producer species implying fratricidal effects, and impacts on antibiotic susceptibility. As such, these peptides prove of interest for further studies in terms of their mechanism and impact on cellular processes The discovery of the inhibitory activity of RaS-RiPPs such as tryglysins, streptosactins, enteropeptins, threoglucins and bicyclostreptins, ^33^ ^,^ ^46^ ^,^ ^47^ ^,^ ^49^ also implies their importance for interbacterial competition in communities. With the presence of biosynthetic gene clusters in multiple species within most of the 16 subfamilies, ^26^ it presents the possibility that additional unidentified RaS-RiPP products might exist. With much more to uncover regarding streptococcal small peptides and RaS-RiPP mature products, this review presents an in-depth summary of our current knowledge today and provides insight for future research.

## Data Availability

No data is associated with this article.
