# Supplementary material for: Rgg/SHP transcriptional regulators, RaS-RiPPs, and their impacts in streptococci
Source: F1000Res. 2026 Jun 3;15:416. Originally published 2026 Mar 19. [Version 2] doi: 10.12688/f1000research.178446.2 (PMC13361665; doi:10.12688/f1000research.178446.2)
Supplement: Peer review history

# Reviewer report: Reviewer response for version 2

**DOI:** 10.5256/f1000research.200787.r490737

**Published:** 2026-06-09

**Authors:** Rozenn Gardan, Quentin Caillot

**Affiliations:** 1 AgroParisTech (Ringgold ID: 84338), Paris, Île-de-France, France; 2 Institut National de Recherche pour l'Agriculture l'Alimentation et l'Environnement Centre Ile-de-France Jouy-en-Josas Antony (Ringgold ID: 74288), Jouy-en-Josas, Île-de-France, France; 3 Paris-Saclay University, Gif-sur-Yvette, Île-de-France, France; 4 INRAE, Paris-Saclay University, Jouy-en-Josas, France

Dear Author,

 We consider that the authors have addressed all the concerns we raised in our peer review report.

 We agree with the authors that no reference as yet demonstrated a direct link between Rgg9420, RggC, and streptosactin production.

 We thank the authors for comparing the amino acid sequences of the various regulators.

Is the review written in accessible language?

Yes

Are all factual statements correct and adequately supported by citations?

Partly

Are the conclusions drawn appropriate in the context of the current research literature?

Yes

Is the topic of the review discussed comprehensively in the context of the current literature?

Yes

Reviewer Expertise:

NA

We confirm that we have read this submission and believe that we have an appropriate level of expertise to confirm that it is of an acceptable scientific standard.

# Reviewer report: Reviewer response for version 1

**DOI:** 10.5256/f1000research.196831.r469296

**Published:** 2026-04-13

**Authors:** Reid Wilkening

**Affiliations:** 1 University of Colorado, Aurora, Colorado, USA

Overall this is a thorough review of Rgg/Shp signaling and RaS-RIPP peptides in the Streptococci. The article begins by explaining the core concepts of Rgg/Shp QS, then moves into a species-by-species explanation of Rgg/Shp QS in numerous streptococcal species. It next moves to a discussion of the role of QS in competence regulation, before closing with a discussion of the Ras-RIPP peptides. 

 The review is overall quite thorough, with appropriate citations and generally helpful figures. However, it could benefit from some thoughtful restructuring to improve clarity and reduce redundancy. I would encourage the authors to spend time considering the order in which the species are presented. As it stands, it is hard to follow a through-line from one species to the next. In addition, several less-common streptococci. e.g. Strep ferus receive a longer treatment than more clinically relevant species such as GBS. Introducing S. gordonii as the first species may help frame the discussion, and some comments orienting the reader to what will come next would be nice. There are also several places were redundant explanations creep in, As the QS pathways are well conserved, the article could be made more concise and impactful by ensuring that each point is necessary. I also question the placement of the discussion of natural competence in Streptococci so distant from the discussion of Strep pneumonia. The figure here perhaps could also be modified to better illustrate the similarities and differences between the S mutant and S. pneumonia. 

 Finally, the section of the Ras-Ripps is interesting, but would be more helpful by helping orient the reader to the various subfamilies in a different way. I was getting a bit lost in the 3- and 4-letter nomenclature. Figure 4 could also be modified to be more impactful, the right of the figure adds little at the moment. Perhaps the authors could highlight how the Rggs interact with the Rass promoters, as well as give examples of proposed functions of the various Ripps.

Is the review written in accessible language?

Partly

Are all factual statements correct and adequately supported by citations?

Yes

Are the conclusions drawn appropriate in the context of the current research literature?

Yes

Is the topic of the review discussed comprehensively in the context of the current literature?

Yes

Reviewer Expertise:

Microbiology, medicine.

I confirm that I have read this submission and believe that I have an appropriate level of expertise to confirm that it is of an acceptable scientific standard, however I have significant reservations, as outlined above.

# Response

**Published:** 2026-05-01

**Authors:** Britta Rued

**Affiliations:** Vet. Micro. & Prevent. Med., Iowa State University College of Veterinary Medicine, Ames, Iowa, USA

*Overall this is a thorough review of Rgg/Shp signaling and RaS-RIPP peptides in the Streptococci. The article begins by explaining the core concepts of Rgg/Shp QS, then moves into a species-by-species explanation of Rgg/Shp QS in numerous streptococcal species. It next moves to a discussion of the role of QS in competence regulation, before closing with a discussion of the Ras-RIPP peptides.*

*The review is overall quite thorough, with appropriate citations and generally helpful figures. However, it could benefit from some thoughtful restructuring to improve clarity and reduce redundancy. I would encourage the authors to spend time considering the order in which the species are presented. As it stands, it is hard to follow a through-line from one species to the next. In addition, several less-common streptococci. e.g. Strep ferus receive a longer treatment than more clinically relevant species such as GBS. Introducing S. gordonii as the first species may help frame the discussion, and some comments orienting the reader to what will come next would be nice. There are also several places were redundant explanations creep in, As the QS pathways are well conserved, the article could be made more concise and impactful by ensuring that each point is necessary. I also question the placement of the discussion of natural competence in Streptococci so distant from the discussion of Strep pneumonia. The figure here perhaps could also be modified to better illustrate the similarities and differences between the S mutant and S. pneumonia.*

 Thank you for your comments. We agree that the article could benefit from restructuring. We acknowledge that the reviewer suggested placement of *S. gordonii* to be first. Although the first demonstration of Rggs to be transcriptional regulators were found in this species, *S. gordonii *was not the first streptococcal species to be demonstrated to use Rgg/SHP systems in quorum sensing. Given the changes in structure requested by another reviewer, we have attempted to rearrange these in a way that is amenable to both requested modifications. Thus, we have changed review to discuss *S. thermophilus *and *S. pyogenes *first (given they were the first species demonstrated to use Rgg/SHP systems in QS), *S. pneumonia*e, then *S. gordonii*.

 Concerning the longer discussion of *S. ferus* over GBS, we have added additional text examining GBS and Rgg/SHP QS systems in this species and renamed the section on “other streptococci” to “ *Streptococcus agalactiae* and other streptococci”.

 Concerning framing the discussion and introduction of streptococcal species to be discussed, we have added a section in the introduction to better orient the reader and have attempted to better introduce these topics.

 Additionally, we have edited the text to avoid redundancy between different sections (eg. eliminating the restating of RaS-RiPP definition and other repeated explanations). We have also changed our explanations of QS pathways to make these more concise and clearer.

 Concerning the placement of the section on competence on *S. pneumoniae*, we agree with the reviewer and note other reviewers had the same feedback. We have moved this discussion to the section on competence and have modified the figure and figure legend on competence (now Figure 4), to help better illustrate the differences and similarities between signaling via CSP in *S. pneumoniae* and signaling via XIP in *S. mutans.*

*Finally, the section of the Ras-Ripps is interesting, but would be more helpful by helping orient the reader to the various subfamilies in a different way. I was getting a bit lost in the 3- and 4-letter nomenclature. Figure 4 could also be modified to be more impactful, the right of the figure adds little at the moment. Perhaps the authors could highlight how the Rggs interact with the Rass promoters, as well as give examples of proposed functions of the various Ripps.*

 Thank you for your comments. We have modified the RaS-RiPP section by restructuring the text to be organized by subfamily. We have also modified the text to include the subfamily name when discussing RiPP products to guide the reader.  We believe this will make the section clearer as we cannot eliminate the nomenclature for RaS-RiPP subfamilies.

 We have modified Figure 4 (now Figure 6) to include the modifications installed by the RaS-RiPP operons and condensed the structures to help better summarize the various subfamilies as the reviewer suggests. Concerning the comment on highlighting how Rggs interact with RaS-promoters, we have added an arrow to Figure 7 indicating that current data support the hypothesis that Rgg transcriptional regulators directly regulate RaS-RiPPs at the promoter level. However, we should note that this has only been directly demonstrated for the RaS-RiPP tryglysin in *S. mutans *(Rued et al., 2021 mBio) and demonstrated in *S. thermophilus* where SHP peptide production and processing was tied to RaS-RiPP levels in *S. thermophilus *(Caillot et al., 2025. J. Bac) *. *Therefore, we hesitate to make overarching conclusions on how Rggs exactly function to control RaS-RiPP induction, given the large breadth of these systems in streptococci. Known functions of RaS-RiPPs are summarized in Table 2, but the majority of RaS-RiPPs have not been evaluated for function in any way.

# Reviewer report: Reviewer response for version 1

**DOI:** 10.5256/f1000research.196831.r469299

**Published:** 2026-04-09

**Authors:** Rozenn Gardan, Quentin Caillot

**Affiliations:** 1 AgroParisTech (Ringgold ID: 84338), Paris, Île-de-France, France; 2 Institut National de Recherche pour l'Agriculture l'Alimentation et l'Environnement Centre Ile-de-France Jouy-en-Josas Antony (Ringgold ID: 74288), Jouy-en-Josas, Île-de-France, France; 3 Paris-Saclay University, Gif-sur-Yvette, Île-de-France, France; 4 INRAE, Paris-Saclay University, Jouy-en-Josas, France

The review, "Rgg/SHP transcriptional regulators, RaS-RiPPs, and their impacts in streptococci," begins with an exhaustive presentation of the SHP/Rgg systems found in streptococci. The systems that have been studied experimentally are described according to their species. The mechanisms, including the partners involved in the life cycle of SHP pheromones, are depicted, as are the functions controlled by these systems. The first table summarizes this information and includes the amino acid sequences of the pheromones and the names of representative strains. Next, the two mechanisms involved in inducing natural competence in streptococci are described. The final section of the review is dedicated to RaS-RiPPs (Radical S-adenosylmethionine enzyme Ribosomally translated and Post-translationally modified Peptides). The operons whose expression leads to the production of these modified peptides are the primary targets of the SHP/Rgg systems in streptococci. This section presents the chemistry, structure and functions of these RaS-RiPPs in streptococci. A second table provides an exhaustive overview of these data.

 This review is clear and sound. For the first time, it summarizes the different SHP/Rgg systems in streptococci. Interestingly, the timeline of the discovery of some SHP/Rgg systems is also provided. The review emphasizes the link between the SHP/Rgg systems and the RaS-RiPPs and includes useful tables. This approach has never been done with such precision, providing a useful overview of the SHP/Rgg systems, the RaS-RiPPs, and their connections.

**Major comments**

 - At the beginning of the introduction, quorum sensing in Gram-positive bacteria appears to be limited to the RRNPP superfamily.  Two-component systems should also be mentioned. Members of the RRNPP superfamily are not united by the fact that they are transcriptional regulators that respond to a small autoinducer. Rap are not transcriptional regulators, they are phosphatases or proteins that interact with regulators. Members of this superfamily are united by a conserved TPR domain architecture characterized by 6 to 9 TPR motif repeats in their C-terminal part (Felipe-Ruiz et al., 2022 [DOI 10.1128/mbio.02514-22]) that respond to a small peptide autoinducer. Members of this superfamily that are transcriptional regulators have an additional HTH domain in their N-terminal part. In conclusion, the beginning of the introduction needs rewriting.

 - In the section ‘A brief overview of natural competence in streptococci’; sigX of *S. mutans* is described as a gene whose expression can be directly regulated by ComE-P, as demonstrated in *S. pneumoniae*. The expression of sigX is only directly controlled by ComRS in *S. mutans*. ComCDE is involved in the production of bacteriocin. See ref 44 and (Reck et al.,2015).

**Minor comments**

Introduction

 Paragraph1. 

 - The second and third sentences : 'Chemical signals' appear to differ from 'autoinducers' or 'pheromones'. Furthermore, the detection of these signals is presented before their production. These points could be clarified.

 - Instead of ‘small inducer ref 12-14’, ‘peptide’ with ref 12 and 16 would be more relevant

 - ‘bind to genes’ : replace ‘gene’ with ‘promoter’

 - Ref. 17 is related to ComR, which is no longer considered a Rgg regulator [Talagas et al.,2016] and as stated in ‘brief overview of natural competence in streptococci’

 Paragraph2. 

 - ‘The transcriptional regulation of these systems varies from species to species’ : Could you be more specific?

 - ’Of these targets, RaS-RiPPs…’ should be replaced with ‘Operons involved in the production of RaS-RiPP’

 - ’They have been shown to inhibit other streptococcal species and have varying effects on antibiotic resistance and growth’. Only some of them have been shown to inhibit other streptococcal species…

*Streptococcus pneumoniae*

 - The first paragraph would be more relevant in the section entitled 'A brief overview of natural competence in streptococci'. In this paragraph replace ‘activation of the alternative sigma factor’ with ‘production of…’ and ‘production of genes’ with ‘expression of genes’

 - Second paragraph: there is a positive feedback loop because the *shp* gene is one of the targets of the Rgg/SHP939 system. This could be explained in more detail.

*Streptococcus pyogenes*

 - First paragraph: Opp imports the mature SHP (as stated in the following sentence), PptAB exports the precursor. The membrane protease Eep (and not the *eep *gene) matures the SHP precursor.

*Streptococcus mutans*

 - ‘Like other Rgg/SHP systems, induction of the RaS-RiPP relies on the presence of Rgg and SHP and requires the proteins PptAB for SHP import and OppD for SHP export’: The roles of PptAB and Opp have been inverted. Furthermore, only the OppD subunit is mentioned here; for consistency, it should be written as Opp or OppABCD. 

*Streptococcus thermophilus*

 - First paragraph: replace ‘controls the expression of another peptide’ with ‘controls the expression of an operon involved in the production of another peptide called Pep1357c…’ Next sentence ‘an efflux transporter that matured..’: we still don’t know how the streptide is matured.

 - ‘This included Rgg1299/SHP1299 (Table 1), which was demonstrated to function as a Rgg/SHP system, although the function of its gene targets is unknown,25,89 Rgg9420/SHP279 and Rgg7530/SHP273, although these identified SHPs were not expressed under experimental conditions.’ This is only true for SHP273. SHP279 is indeed secreted, although it is weakly expressed. This is demonstrated in ref 37. Additionally, this study demonstrated that all SHPs are secreted and re-imported simultaneously. 

 - second paragraph: ‘Again, these proteins have various loci at which they target for regulation’ Which proteins? Is ComR included in the sentence ? RggC is an outdated name for Rgg9420 and is therefore primarily responsible for producing streptosactine.

 - Replace ‘Finally, several *S. thermophilus* strains (JIM8232, CNRZ1066)’ with   ‘Finally, several *S. thermophilus* strains  including JIM8232, CNRZ1066’ Otherwise it suggests that only these two strains produce these RaS-RiPPs.

Other streptococci

 - Second paragraph: for inter-species cross talk reference add: (Cook L et al.,2013).

RaS-RiPPs as natural products and targets of Rgg/SHP QS

 - Second paragraph: a definition for RaS-RiPP is already provided in the previous paragraph and in the introduction.

 -Replace ‘During RiPP biosynthesis, a precursor peptide composed of a N-terminal region…’ with ‘During RaS-RiPP biosynthesis, a precursor peptide composed of a N-terminal region…’

RaS-RiPPs’ and Rgg/SHP interplay

 - This section could be shortened since the discovery of the sixteen families is already described in the 'RaS-RiPPs as Natural Products and Targets of Rgg/SHP QS' section.

Table 1

 Column 2: specify SHP/XIP/LCP due to the presence of RopB.

Table 2

 Enteropeptins: could you specify the different forms (A, B, C, and D ) to remain consistent with bicyclostreptin and tryglysin. Also specify which forms exhibit growth inhibition against *E. cecorum*.

 Bicyclostreptin: “t” letter is missing in all four forms in the table. 

Figure 1

 - Maintain consistent color nomenclature between the mature peptide secreted by PptAB and the re-imported peptide via Opp. Since the sequence does not change, the color should not change either.

 - The Rgg-box/promoter depiction should be explained in the figure legend. 

Figure 2

 - Gene “SigX” should be written “ *sigX*.” in the arrow

 - The meaning of the different type of arrow should be specify in the legend

 - The CSP part should eventually be modified after the bibliography is checked (see the major comment).

Figure 3

 - Either display all three enteropeptin forms as done for bicyclostreptin and tryglysin, or show only one form for the three RaS-RiPPs for consistency.

 - Add Ryptide to  “RRR”  . 

Figure 4

 - Same comment regarding color code consistency for the SHP precursor and mature form.

Is the review written in accessible language?

Yes

Are all factual statements correct and adequately supported by citations?

Partly

Are the conclusions drawn appropriate in the context of the current research literature?

Yes

Is the topic of the review discussed comprehensively in the context of the current literature?

Yes

Reviewer Expertise:

NA

We confirm that we have read this submission and believe that we have an appropriate level of expertise to confirm that it is of an acceptable scientific standard, however we have significant reservations, as outlined above.

## References

[1] : Structural and Genomic Evolution of RRNPPA Systems and Their Pheromone Signaling. *mBio* .2022;13(6) : 10.1128/mbio.02514-22 10.1128/mbio.02514-22 PMC976570936259720

[2] : The Alternative Sigma Factor SigX Controls Bacteriocin Synthesis and Competence, the Two Quorum Sensing Regulated Traits in Streptococcus mutans. *PLOS Genetics* .2015;11(7) : 10.1371/journal.pgen.1005353 10.1371/journal.pgen.1005353 PMC449767526158727

[3] : Structural Insights into Streptococcal Competence Regulation by the Cell-to-Cell Communication System ComRS. *PLOS Pathogens* .2016;12(12) : 10.1371/journal.ppat.1005980 10.1371/journal.ppat.1005980 PMC513189127907189

[4] : Interspecies Communication among Commensal and Pathogenic Streptococci. *mBio* .2013;4(4) : 10.1128/mBio.00382-13 10.1128/mBio.00382-13 PMC373518423882015

# Response

**Published:** 2026-05-01

**Authors:** Britta Rued

**Affiliations:** Vet. Micro. & Prevent. Med., Iowa State University College of Veterinary Medicine, Ames, Iowa, USA

*The review, "Rgg/SHP transcriptional regulators, RaS-RiPPs, and their impacts in streptococci," begins with an exhaustive presentation of the SHP/Rgg systems found in streptococci. The systems that have been studied experimentally are described according to their species. The mechanisms, including the partners involved in the life cycle of SHP pheromones, are depicted, as are the functions controlled by these systems. The first table summarizes this information and includes the amino acid sequences of the pheromones and the names of representative strains. Next, the two mechanisms involved in inducing natural competence in streptococci are described. The final section of the review is dedicated to RaS-RiPPs (Radical S-adenosylmethionine enzyme Ribosomally translated and Post-translationally modified Peptides). The operons whose expression leads to the production of these modified peptides are the primary targets of the SHP/Rgg systems in streptococci. This section presents the chemistry, structure and functions of these RaS-RiPPs in streptococci. A second table provides an exhaustive overview of these data.*

*This review is clear and sound. For the first time, it summarizes the different SHP/Rgg systems in streptococci. Interestingly, the timeline of the discovery of some SHP/Rgg systems is also provided. The review emphasizes the link between the SHP/Rgg systems and the RaS-RiPPs and includes useful tables. This approach has never been done with such precision, providing a useful overview of the SHP/Rgg systems, the RaS-RiPPs, and their connections.*

 First, we thank the reviewer for their recognition of the work that has gone into preparing the manuscript. Second, we greatly appreciate the thorough review provided; we believe this has made the review much stronger and have made changes to the manuscript as the reviewer has suggested, see each reply below.

*Major comments*

*- At the beginning of the introduction, quorum sensing in Gram-positive bacteria appears to be limited to the RRNPP superfamily.  Two-component systems should also be mentioned. Members of the RRNPP superfamily are not united by the fact that they are transcriptional regulators that respond to a small autoinducer. Rap are not transcriptional regulators, they are phosphatases or proteins that interact with regulators. Members of this superfamily are united by a conserved TPR domain architecture characterized by 6 to 9 TPR motif repeats in their C-terminal part (Felipe-Ruiz et al., 2022 [DOI 10.1128/mbio.02514-22]) that respond to a small peptide autoinducer. Members of this superfamily that are transcriptional regulators have an additional HTH domain in their N-terminal part. In conclusion, the beginning of the introduction needs rewriting.*

 Thank you for this important suggestion. We agree that the original text did not include all working mechanisms of quorum sensing systems in Gram-positive bacteria – although we later discuss ComCDE (a two-component system) in the text. We have now revised the first part of the introduction as suggested that include a brief discussion of both two-component signal transduction systems and RRNPP superfamily members. We also include the statement that RRNPR regulators are united by the conserved TPR domain architecture, and that Rap proteins are phosphatases or proteins that interact with response regulators to modulate their function, as describe by Felipe-Ruiz et al., 2022.

*- In the section ‘A brief overview of natural competence in streptococci’; sigX of S. mutans is described as a gene whose expression can be directly regulated by ComE-P, as demonstrated in S. pneumoniae. The expression of sigX is only directly controlled by ComRS in S. mutans. ComCDE is involved in the production of bacteriocin. See ref 44 and (Reck et al.,2015).*

 You are correct in this assessment, and we have updated this section to correctly reflect the signaling cascade in *S. mutans*. We have clarified that ComE-P does not directly regulate *sigX* in *S. mutans, *but that activation of competence is controlled by ComRS. We emphasize that ComCDE in *S. mutans *is primarily involved in inducing bacteriocin expression, referring to the following references (Son et al., 2015; Reck et al., 2015). We do note that Perry et al., 2009 Mol Micro found that addition CSP of does result in induction of *sigX* (alternatively called *comX*) under specific conditions. However, this induction is indirect as a result of cross-talk between the ComCDE system and ComRS, not via direct activation by ComE-P, as you correctly state and as reported via Underhill et al., 2019, Mol Micro and Underhill et al. 2018, mSphere. We also note this induction is media dependent. This has now been clarified in the text. We instead characterize the ComCDE system in *S. pneumoniae*, where this system induces competence as we illustrate in the updated figure (Now Figure 4).

*Minor comments*

*Introduction*

*Paragraph1.*

*- The second and third sentences : 'Chemical signals' appear to differ from 'autoinducers' or 'pheromones'. Furthermore, the detection of these signals is presented before their production. These points could be clarified.*

  We agree that the sequence of chemical signals and autoinducers were unclear in the original text. We have revised these sentences to better define chemical signals as autoinducers or pheromones. This presents the quorum sensing process starting with signal production followed by release and detection of chemical signals (i.e. autoinducers or pheromones) by cognate receptors to regulate cell-cell communication.

*- Instead of ‘small inducer ref 12-14’, ‘peptide’ with ref 12 and 16 would be more relevant*

 Thank you for this comment. We have made the suggested changes in the text.

*- ‘bind to genes’ : replace ‘gene’ with ‘promoter’*

 We have changed the language in the text.

*- Ref. 17 is related to ComR, which is no longer considered a Rgg regulator [Talagas et al.,2016] and as stated in ‘brief overview of natural competence in streptococci’*

 Thank you for catching this. We have removed reference 17 from this section and have added the provided article to the ‘brief overview of natural competence in streptococci’ section.

*Paragraph2.*

*- ‘The transcriptional regulation of these systems varies from species to species’ : Could you be more specific?*

 Thank you for your suggestion. We have revised the section to clarify that Rgg/SHP systems are often species-specific and target unique loci depending on the streptococcal organism they are present in. We list examples of Rgg/SHP regulated phenotypes across streptococcal species, such as: biofilm formation, colonization, immunomodulatory activities, and virulence in different streptococcal species, and that this is a result of different gene targets being modulated by Rgg/SHP systems.

*- ’Of these targets, RaS-RiPPs…’ should be replaced with ‘Operons involved in the production of RaS-RiPP"*

 We have changed the language in the text.

*- ’They have been shown to inhibit other streptococcal species and have varying effects on antibiotic resistance and growth’. Only some of them have been shown to inhibit other streptococcal species…*

 Thank you for catching this overlook. This has been changed to ‘Some classes of RaS-RiPPs have been shown to inhibit other streptococcal species and have varying effects on antibiotic resistance and growth’.

*Streptococcus pneumoniae*

*- The first paragraph would be more relevant in the section entitled 'A brief overview of natural competence in streptococci'. In this paragraph replace ‘activation of the alternative sigma factor’ with ‘production of…’ and ‘production of genes’ with ‘expression of genes'*

 We agree with the reviewer and have moved this section to the section on “A brief overview of natural competence” as they suggest. Additionally, we have modified the sentence about *sigX* with the suggested language.  

*- Second paragraph: there is a positive feedback loop because the shp gene is one of the targets of the Rgg/SHP939 system. This could be explained in more detail.*

 Thank you for this comment. We agree with the reviewer that our explanation of the Rgg/SHP939 system needed improvement. We have altered the text to explicitly state how the positive feedback loop functions through the upregulation of *shp*.

*Streptococcus pyogenes*

*- First paragraph: Opp imports the mature SHP (as stated in the following sentence), PptAB exports the precursor. The membrane protease Eep (and not the eep gene) matures the SHP precursor.*

 Thank you for this comment. We have altered the text to the following: ‘In this system, SHP pheromones (DI[I/L]IIVGG) require PptAB to export the SHP precursor, a metalloprotease (Eep) to enzymatically mature the precursor peptide, and a functional oligopeptide permease (Opp) transporter to  import the mature SHP.’

 Please see additional comments further addressing requested edits.

# Response

**Published:** 2026-05-01

**Authors:** Britta Rued

**Affiliations:** Vet. Micro. & Prevent. Med., Iowa State University College of Veterinary Medicine, Ames, Iowa, USA

Continued from previous comments addressing edits.

*Streptococcus mutans*

*- ‘Like other Rgg/SHP systems, induction of the RaS-RiPP relies on the presence of Rgg and SHP and requires the proteins PptAB for SHP import and OppD for SHP export’: The roles of PptAB and Opp have been inverted. Furthermore, only the OppD subunit is mentioned here; for consistency, it should be written as Opp or OppABCD.*

 The language has now been modified to Opp to represent the entire complex and the correct protein has been assigned to import/export, as we discuss later in the text and in figures.

*Streptococcus thermophilus*

*- First paragraph: replace ‘controls the expression of another peptide’ with ‘controls the expression of an operon involved in the production of another peptide called Pep1357c…’ Next sentence ‘an efflux transporter that matured..’: we still don’t know how the streptide is matured.*

 We have modified the first sentence with the language you suggested. The next sentence regarding streptide maturation has been changed to the following: “Streptide relies on a radical SAM enzyme a (StrB) to generate a mature 9mer product, and an efflux transporter to secrete the peptide outside of the cell. However, the exact mechanism of streptide processing during secretion is still unknown.”

*- ‘This included Rgg1299/SHP1299 (Table 1), which was demonstrated to function as a Rgg/SHP system, although the function of its gene targets is unknown,25,89 Rgg9420/SHP279 and Rgg7530/SHP273, although these identified SHPs were not expressed under experimental conditions.’*

*This is only true for SHP273. SHP279 is indeed secreted, although it is weakly expressed. This is demonstrated in ref 37. Additionally, this study demonstrated that all SHPs are secreted and re-imported simultaneously.*

 We have reworded the text to correctly reflect that *shp279 *is weakly expressed and *shp273 *did not show any effect under the experimental conditions tested. We have also incorporated that SHPs are secreted and are subsequently reimported as typically seen in the role of intracellular quorum sensing systems.

*- second paragraph: ‘Again, these proteins have various loci at which they target for regulation’ Which proteins? Is ComR included in the sentence? RggC is an outdated name for Rgg9420 and is therefore primarily responsible for producing streptosactine.*

 We agree that the original wording was unclear and have revised the text to explicitly refer to Rgg regulators. We clarify that the ComRS system in *S. thermophilus* involves ComR, an Rgg-like transcriptional regulator, and ComS, its associated signaling peptide (Gardan et al., 2013, *J Bacteriol*). To improve clarity and flow, we have moved the ComRS discussion to the competence section, where competence-related systems across species are discussed together.

 Regarding Rgg9420, we could not locate in the literature where streptosactin production has been linked to this Rgg. Caillot et al., 2025 ( *J Bacteriol)* reported that another Rgg/SHP system (Rgg1358 or SHP/Rgg _S_Thermo13_) is involved in producing streptosactin and regulating a similar RiPP-associated pathway, but we did not observe Rgg9420 as being reported to produce streptosactin in this publication. Earlier work by Fernandez et al., 2006, ( *Archives of Microbiology)* describes the *rggC *( *rggC1 and rggC2*) locus with a frameshift mutation and encoding Rgg-like proteins. Upon performing a clustal BLAST protein alignment between RggC2, RggC1 and Rgg9420, there is a high amount of conservation between the three amino acid sequences, but noted several aspects: RggC1 comprised an N-terminal portion of Rgg9420, which overlapped with the start of RggC2, RggC2 contained the rest of Rgg9420, but also possessed several amino acid changes in the portion it shared with Rgg9420, indicating that while there were shared sequences RggC1/RggC2 was not identical to Rgg9420. Nevertheless, to examine if Rgg9420 was associated with streptosactin, we looked at the LMD-9 locus surrounding this Rgg and found that it does indeed encode for a streptosactin biosynthetic gene locus as the reviewer mentions – but we could not locate literature directly demonstrating that streptosactin is regulated by Rgg9420. We therefore still describe the *rggC* locus as being associated with oxidative stress, but would appreciate any additional references the reviewer may suggest demonstrating a direct link between Rgg9420, RggC, and streptosactin production that we may have missed.

*- Replace ‘Finally, several S. thermophilus strains (JIM8232, CNRZ1066)’ with   ‘Finally, several S. thermophilus strains  including JIM8232, CNRZ1066’ Otherwise it suggests that only these two strains produce these RaS-RiPPs.*

 We have changed the sentence to the following ‘ *S. thermophilus* strains, including JIM8232 and CNRZ1066, use Rgg/SHP systems to control the production of downstream RaS-RiPPs.’

*Other streptococci*

*- Second paragraph: for inter-species cross talk reference add: (Cook L et al.,2013).*

 We have referenced the suggested article in the text.

*RaS-RiPPs as natural products and targets of Rgg/SHP QS*

*- Second paragraph: a definition for RaS-RiPP is already provided in the previous paragraph and in the introduction.*

 We have eliminated the sentence ‘This class of natural products detailed here are called RaS-RiPPs, a specific subtype of RiPPs that post-translationally modified by RaS-enzymes.’ to avoid redundancy.

*-Replace ‘During RiPP biosynthesis, a precursor peptide composed of a N-terminal region…’ with ‘During RaS-RiPP biosynthesis, a precursor peptide composed of a N-terminal region…’*

 The sentence has now been modified as the reviewer suggests.

*RaS-RiPPs’ and Rgg/SHP interplay*

*- This section could be shortened since the discovery of the sixteen families is already described in the 'RaS-RiPPs as Natural Products and Targets of Rgg/SHP QS' section.*

 We have attempted to shorten the section as the reviewer suggests, we have removed sentences regarding the discovery of the 16 subfamilies and gene cluster bioinformatic search.

*Table 1*

*Column 2: specify SHP/XIP/LCP due to the presence of RopB.*

 We have modified this as the reviewer suggests and note that several other LCPs have been shown to be functional. These are now included in the table as well.

*Table 2*

*Enteropeptins: could you specify the different forms (A, B, C, and D ) to remain consistent with bicyclostreptin and tryglysin. Also specify which forms exhibit growth inhibition against E. cecorum.*

 We have done as the reviewer suggests, and specified which forms inhibit growth inhibition against *E. cecorum *(to our knowledge, this has only been documented for purified Enteropeptin A).

*Bicyclostreptin: “t” letter is missing in all four forms in the table.*

 Thank you for the catch. We have corrected this.

 Please see additional comments addressing the requested edits.

# Response

**Published:** 2026-05-01

**Authors:** Britta Rued

**Affiliations:** Vet. Micro. & Prevent. Med., Iowa State University College of Veterinary Medicine, Ames, Iowa, USA

Please see additional comments addressing requested edits. 

*Figure 1*

*- Maintain consistent color nomenclature between the mature peptide secreted by PptAB and the re-imported peptide via Opp. Since the sequence does not change, the color should not change either.*

 We have corrected this as the reviewer suggests. Please note that this figure is now Figure 2 (per adjustments requested by other reviewers).

*- The Rgg-box/promoter depiction should be explained in the figure legend.*

 We have explained this in the updated figure legend and updated the figure to better reflect the Rgg-box/promoter orientation. Again, please note that this figure is now Figure 2 (per adjustments requested by other reviewers).

*Figure 2*

*- Gene “SigX” should be written “sigX.” in the arrow*

 Thank you for the catch, we have corrected this. Please note that this figure is now Figure 4, per additions requested by other reviewers.

*- The meaning of the different type of arrow should be specify in the legend*

 We have added clarification concerning this to the legend, as the reviewer suggests, and changed colors to help better clarify what each arrow means. Larger black arrows indicate induction of a protein/peptide or regulation by a protein or protein complex. Small black arrows located on gene diagrams indicate promoters. Light blue arrows indicate transfer of a phosphate (specifically from ComD to ComE). Light gray arrows indicate processing of a peptide or movement of a peptide through a protein complex. Please note that this figure is now Figure 4, per additions requested by other reviewers.

*- The CSP part should eventually be modified after the bibliography is checked (see the major comment).*

 We agree with the reviewer, and we have modified this figure instead to clarify that the CSP portion reflect competence as it is carried out in *S. pneumoniae*, while the XIP portion reflect induction of competence in *S. mutans*. We have also corrected the text that refers to this figure. Please note that this figure is now Figure 4, per additions requested by other reviewers.

*Figure 3*

*- Either display all three enteropeptin forms as done for bicyclostreptin and tryglysin, or show only one form for the three RaS-RiPPs for consistency.*

 As the reviewer suggests, we have modified this figure so that only one structure for each RaS-RiPP family is shown. We now show Tryglysin A, Bicyclostrepin A, and the Enteropeptin core structure (modifications that differentiate Enteropeptins A, B, and C are outside of this core structure). We have also indicated the bonds installed by RaS and additional modification enzymes encoded by RaS-RiPP operons are shown in red, to incorporate comments from another reviewer. Please note that this figure is now Figure 6, per additions requested by other reviewers.

*- Add Ryptide to  “RRR”  .*

 Added to figure as requested. Please note that this figure is now Figure 6, per additions requested by other reviewers.

*Figure 4*

*- Same comment regarding color code consistency for the SHP precursor and mature form.*

 We have changed this as the reviewer has requested, to keep color consistency. Please note that this figure is now Figure 7, per additions requested by other reviewers.

# Reviewer report: Reviewer response for version 1

**DOI:** 10.5256/f1000research.196831.r469297

**Published:** 2026-04-08

**Authors:** Laura Cook

**Affiliations:** 1 Binghamton University, Binghamton, New York, USA

This is a nicely written and thorough review of the Rgg/SHP and RaS-RiPP systems in streptococci. Some of the formatting and emphasis could be changed to make the information clearer but overall, this is a well-done review. Changes I would suggest:

1. The organization could be altered a bit. You start with S. pneumo but refer a lot to thermophilus and GAS being the organisms with the earliest studies on these systems. It would make more sense to me to put S. thermophilus first followed by GAS then pneumo.
2. In terms of organization, there is a lot here on the competence systems. This is a bit confusing as it is stated that ComCDE and ComRS systems are not Rgg/SHP system. ComR is not mentioned until page 8 but ComCDE is discussed very early. It is not really clear how they link to the Rggs and so much emphasis on competence without a clear link is confusing, especially as you say the are distinct from the Rgg/SHP systems yet have a section and a figure about them.
3. Additional figures could help clarify in a few places. A figure could help with the Group I versus Group II SHPs  in the introduction. A figure on the general chemical reaction for the RaS-RiPPs on page 10 could also be helpful.
4. You list Figure 3 throughout describing the structures and modifications but Figure 3 doesn’t really show anything except the chemical structures so referring to it may not make sense until the end of that section.

Minor edits (difficult without line numbers to describe)

1. Page 4 paragraph 2: Maybe an additional sentence on the crosstalk would be helpful?
2. Page 4 paragraph 3: What do you mean when you say “regulatory nexus”? Also, a figure here on the the Rgg1518 and Rgg939 interaction would also be helpful. There is also a tense change (are and then was).
3. Page 4 paragraph 4: Missing the word “the” in second sentence, wording is off.
4. Page 4 last paragraph: Add citation for “aside from S. thermophilus”
5. Page 6 short paragraph: Tense change (are essential…comprised)
6. Page 7 paragraph 4: It is confusing whether this system has been studied in both NZ131 and M1 and what the citation is for M1 serotype.
7. S. gordonii paragraph: Can you further describe the difference between Rgg and RggD?
8. Page 11 last paragraph capitalize and italicize Streptococcus.
9. Page 13 media is plural, medium is singular.
10.  On page 14 you could mention that the enteropeptins are also made by S. thermophilus.  

Is the review written in accessible language?

Yes

Are all factual statements correct and adequately supported by citations?

Yes

Are the conclusions drawn appropriate in the context of the current research literature?

Yes

Is the topic of the review discussed comprehensively in the context of the current literature?

Yes

Reviewer Expertise:

Microbiology, streptococci

I confirm that I have read this submission and believe that I have an appropriate level of expertise to confirm that it is of an acceptable scientific standard, however I have significant reservations, as outlined above.

# Response

**Published:** 2026-05-01

**Authors:** Britta Rued

**Affiliations:** Vet. Micro. & Prevent. Med., Iowa State University College of Veterinary Medicine, Ames, Iowa, USA

*This is a nicely written and thorough review of the Rgg/SHP and RaS-RiPP systems in streptococci. Some of the formatting and emphasis could be changed to make the information clearer but overall, this is a well-done review. Changes I would suggest: *

- *The organization could be altered a bit. You start with S. pneumo but refer a lot to thermophilus and GAS being the organisms with the earliest studies on these systems. It would make more sense to me to put S. thermophilus first followed by GAS then pneumo.*

**Response: **Thank you for your comment. Upon review of the organization, we highly agree the article could benefit from restructuring. We have moved *S. thermophilus* to the first section followed by *S. pyogenes* and *S. pneumoniae*.

- *In terms of organization, there is a lot here on the competence systems. This is a bit confusing as it is stated that ComCDE and ComRS systems are not Rgg/SHP system. ComR is not mentioned until page 8 but ComCDE is discussed very early. It is not really clear how they link to the Rggs and so much emphasis on competence without a clear link is confusing, especially as you say the are distinct from the Rgg/SHP systems yet have a section and a figure about them.*

**Response: **We agree with the reviewer that there is a mentioning of competence systems that is separate from the ‘brief overview of competence systems’ section of the paper. We have moved some of the discussion of these competence systems to the designated section.

 We have also provided further context on the inclusion of competence systems, from the standpoint that ComRS systems were originally considered to belong to the Rgg/SHP system regulator family, but are now thought to be constitute their own class of regulators. Given the literature and similarities between these two regulatory classes, we included a discussion of the ComRS system in terms of competence. Due to this, we also felt a short discussion of the ComCDE system and the differences between this and the ComRS system were warranted, considering their connection to competence and that these both constitute important peptide signaling systems in streptococci.

- *Additional figures could help clarify in a few places. A figure could help with the Group I versus Group II SHPs in the introduction. A figure on the general chemical reaction for the RaS-RiPPs on page 10 could also be helpful.*

**Response: **We thank the reviewer for the helpful feedback. We have included a Figure on Rgg/SHP Groups and LCPs in the introduction, and further clarified this (Figure 1), based on the original definitions outlined by Fleuchot et al., 2011, Mol. Micro. and Fleuchot et al, 2013, PloS One. For the second figure, while each RaS-RiPP modification is quite specific, we have added a generalized chemical reaction for RaS enzymes (i.e. formation of a radical and use of *S*-adenosylmethonine). This is now included as Figure 5 (general mechanism of RaS-RIPP biosynthesis).

- *You list Figure 3 throughout describing the structures and modifications but Figure 3 doesn’t really show anything except the chemical structures so referring to it may not make sense until the end of that section.*

**Response: **Thank you for this feedback, we have now included the location of the modifications made by RaS enzymes and additional enzymes encoded by their respective RaS-RiPP families in the Figure (now Figure 6). This now matches with referring to this in terms of the modifications installed by these operons.

*Minor edits (difficult without line numbers to describe) *

- *Page 4 paragraph 2: Maybe an additional sentence on the crosstalk would be helpful?*

**Response: **Thank you for the suggestion. We agree that the observed crosstalk between *spd_1513-1517* operon and different Rggs were not clear. We have now added a concluding statement explaining that multiple Rgg regulators influence the function of a shared target operon. This highlights that the Rgg quorum-sensing pathways function as an interconnected regulatory network rather than just independent signaling systems. This further supports coordinated regulation across Rgg/SHP systems.

- *Page 4 paragraph 3: What do you mean when you say “regulatory nexus”? Also, a figure here on the the Rgg1518 and Rgg939 interaction would also be helpful. There is also a tense change (are and then was).*

**Response: **Thank you for the comment – we realize that this interpretation was not clear and have reworded this to better reflect the actual interaction of the Rggs in *S. pneumoniae*. Rgg1518 expression has been reported to be affected by Rgg0939 and Rgg144 (Shlla et al., 2021. Mol. Micro.), and induction of the Rgg/SHP systems controlled by Rgg0939 and Rgg144 require each other’s presence for full induction by their cognate SHPs (Zhi et al., 2018. Sci Reports). These regulators converge on similar processes: sugar metabolism and interactions with the host (either influencing colonization or virulence). We have prepared a figure to help illustrate these concepts better as the reviewer suggests (Now Figure 3). 

- *Page 4 paragraph 4: Missing the word “the” in second sentence, wording is off.*

**Response: **We believe we have corrected this in the text; however, the reviewer’s comments do not correspond to the correct location in the text in our proof version. If this error is still present within the text please let us know.

- *Page 4 last paragraph: Add citation for “aside from S. thermophilus”*

**Response: **We agree that the statement requires appropriate support. We have added appropriate citations to support this statement that describes early characterization of Rgg regulators and pheromone signaling systems including the ComRS system in *S. thermophilus *(Fontaine et al., 2010. J Bacteriol, Fleuchot et al., 2011. Mol Microbiol).

- *Page 6 short paragraph: Tense change (are essential…comprised)*

**Response: **Thank you for catching this inconsistency. The word ‘composed’ has now been changed to ‘compose’.

- *Page 7 paragraph 4: It is confusing whether this system has been studied in both NZ131 and M1 and what the citation is for M1 serotype.*

**Response: **We agree that the original text was unclear regarding the distinction between M1 serotype and NZ131 strain. We have reworded the paragraph and have clearly stated the findings from both the strains, with references cited accordingly. The revised text now clarifies that Rgg2-dependent regulation has been studied in both M1 serotype and NZ131 strain under different genetic backgrounds with distinct observations. These findings suggest that deletion of Rgg2 increases the expression of virulence genes such as *slo, nga*, whereas activation of this system results in the suppression of these genes.

- *S. gordonii paragraph: Can you further describe the difference between Rgg and RggD?*

**Response: **We have expanded the description of the difference between *rgg *and *rggD *genes. They are structurally similar but carry different functions. The gene *rgg *is a positive regulator of *gtfG *expression and glucosyltransferase activity, whereas *rggD *does not have any effect on *gtfG *expression, GTF activity, or in the transcription of adjacent/downstream genes tested under different growth conditions. We also note that the authors suggested *rggD *might regulate a distally located gene rather than the adjacent locus (Vickerman et al., 2001).  

- *Page 11 last paragraph capitalize and italicize Streptococcus.*

**Response: **Thank you for this catch, we have changed this as the reviewer suggests.  

- *Page 13 media is plural, medium is singular.*

**Response: **We have changed this as the reviewer suggests.  

- * On page 14 you could mention that the enteropeptins are also made by S. thermophilus.  *

**Response: **We have reviewed this section within the text and included “Finally, several *S. thermophilus* strains (JIM8232, CNRZ1066) use Rgg/SHP systems to control the production of downstream RaS-RiPPs. These include RaS-RiPPs such as: streptide (SHP/Rgg _gp_sali_6_), streptosactins (SHP/Rgg _Sthermo_13_), bicyclostreptins (SHP/Rgg _gp_sali_4_), enteropeptins (SHP/Rgg _gp_sali_5_), and ryptides (SHP/Rgg _gp_sali_7_) (Table 1)", which describes the production of enteropeptins by *S. thermophilus.*
